# Comparative genome analysis of *Prevotella intermedia* strain isolated from infected root canal reveals features related to pathogenicity and adaptation

**DOI:** 10.1186/s12864-015-1272-3

**Published:** 2015-02-25

**Authors:** Yunfeng Ruan, Lu Shen, Yan Zou, Zhengnan Qi, Jun Yin, Jie Jiang, Liang Guo, Lin He, Zijiang Chen, Zisheng Tang, Shengying Qin

**Affiliations:** Bio-X Institutes, Key Laboratory for the Genetics of Developmental and Neuropsychiatric Disorders(Ministry of Education, Shanghai Jiao Tong University, 1954 Huashang Road, Shanghai, 200030 China; Shanghai Institutes of Pilot Genomics and Human Health, Shanghai, 200030 China; The Fourth Hospital of Jinan City; Taishan Medical College, Jinan, 250031 China; Center for Reproductive Medicine, Shandong Provincial Hospital, Shandong University, Jinan, Shandong China; Department of Endodontics, 9th People’s Hospital, Shanghai JiaoTong University, School of Medicine, Shanghai Key Laboratory of Stomatology, Shanghai, 200011 China

## Abstract

**Background:**

Many species of the genus *Prevotella* are pathogens that cause oral diseases. *Prevotella intermedia* is known to cause various oral disorders e.g. periodontal disease, periapical periodontitis and noma as well as colonize in the respiratory tract and be associated with cystic fibrosis and chronic bronchitis. It is of clinical significance to identify the main drive of its various adaptation and pathogenicity. In order to explore the intra-species genetic differences among strains of *Prevotella intermedia* of different niches, we isolated a strain *Prevotella intermedia* ZT from the infected root canal of a Chinese patient with periapical periodontitis and gained a draft genome sequence. We annotated the genome and compared it with the genomes of other taxa in the genus *Prevotella*.

**Results:**

The raw data set, consisting of approximately 65X-coverage reads, was trimmed and assembled into contigs from which 2165 ORFs were predicted. The comparison of the *Prevotella intermedia* ZT genome sequence with the published genome sequence of *Prevotella intermedia* 17 and *Prevotella intermedia* ATCC25611 revealed that ~14% of the genes were strain-specific. The *Preveotella intermedia* strains share a set of conserved genes contributing to its adaptation and pathogenic and possess strain-specific genes especially those involved in adhesion and secreting bacteriocin. The *Prevotella intermedia* ZT shares similar gene content with other taxa of genus *Prevotella*. The genomes of the genus *Prevotella* is highly dynamic with relative conserved parts: on average, about half of the genes in one *Prevotella* genome were not included in another genome of the different *Prevotella* species. The degree of conservation varied with different pathways: the ability of amino acid biosynthesis varied greatly with species but the pathway of cell wall components biosynthesis were nearly constant. Phylogenetic tree shows that the taxa from different niches are scarcely distributed among clades.

**Conclusions:**

*Prevotella intermedia* ZT belongs to a genus marked with highly dynamic genomes. The specific genes of *Prevotella intermedia* indicate that adhesion, competing with surrounding microbes and horizontal gene transfer are the main drive of the evolution of *Prevotella intermedia*.

**Electronic supplementary material:**

The online version of this article (doi:10.1186/s12864-015-1272-3) contains supplementary material, which is available to authorized users.

## Background

*Prevotella*, previously classified in the genus *Bacteroides*, was a genus of an obligate anaerobic gram-negative rod-shape bacterium. They are classified as “black pigmented bacteria” as they form shiny and smooth colonies with grey, light brown or black color on blood agar plate [1]. Although they generally have a limited ability to ferment amino acid and require hemin and menadione to grow, *Prevotella* is a versatile genus which has been observed in various niches, such as oral cavity, upper respiratory tract, urogenital tract [2], rumen and human feces [3]. Many *Prevotella* taxa from oral cavity are potential/opportunistic pathogens. Gomes *et al* have shown that *Prevotella* spp is associated with pain and swelling and ‘wet’ canal of oral diseases [4-6]. Besides, *Prevotella intermdedia, Prevotella dentalis, Prevotella denticola, Prevotella meleninogenica* are known to be pathogenic under a wide range of situations and invade into the host tissues [7-9].

*Prevotella intermedia*has long been known to be associated with periodontal diseases [10-12], periapical periodontitis [4,5,13] and noma (an acute gangrenous disease) [14,15]. *Prevotella intermedia* also exists at the diseased site of periapical periodontitis [13,16,17] and shows a significantly higher detection ratio in symptomatic sites than in asymptomatic sites [13]. Besides involved in oral diseases, *Prevotella intermedia* was also reported to colonize in the respiratory tract and be associated with cystic fibrosis and chronic bronchitis [6,18]. The association of *Prevotella intermedia* with various diseases raises a question what drives the evolution of *Prevotella intermedia* in different niches. The question also helps us predict how much gene mutation will take for a strain adapt to another niche.

There have been evidences to show that the intra-species difference exists among *Prevotella intermedia* strains in different niches, e.g. the degradative enzyme activity of *Prevotella intermedia* in diseased sites is significantly higher than in healthy sites [19]. However, the difference of the phenotype directly related with pathogenicity could be either genetic or non-genetic such as the regulation of gene expression under different niches and interaction with surrounding microbe. Previous study proved that the intra-species differences of some genes caused different bacterial adaptation to the environment [20,21]. The intra-species variation could be an essential factor to these differences [22]. Therefore, our approach to solve this issue is to assume the drive is genetic factors and explore the intra-species variation among different strains of *Prevotella intermedia*.

The first published genome sequence of *Prevotella intermedia* is that of *Prevotella intermedia* 17. This is a clinical strain isolated from the periodontal pocket and is one of the most studied of the *Prevotella intermedia* strains. The genome has facilitated gene discovery in a number of studies [23-25]. DOE Joint Genome Institute also submitted the draft genome sequence of the type strain, *Prevotella intermedia* ATCC 25611, a strain isolated from empyema. We isolated the clinical strain *Prevotella intermedia* ZT from the infected root canal of a Chinese patient with chronic apical abscess and obtained its draft genome. To further understand the general character of the genus *Prevotella* we also compared this strain with other taxa of the genus *Prevotella*.

## Results and discussion

### The general feature of the *Prevotella intermedia* ZT genome

**Figure 1:**
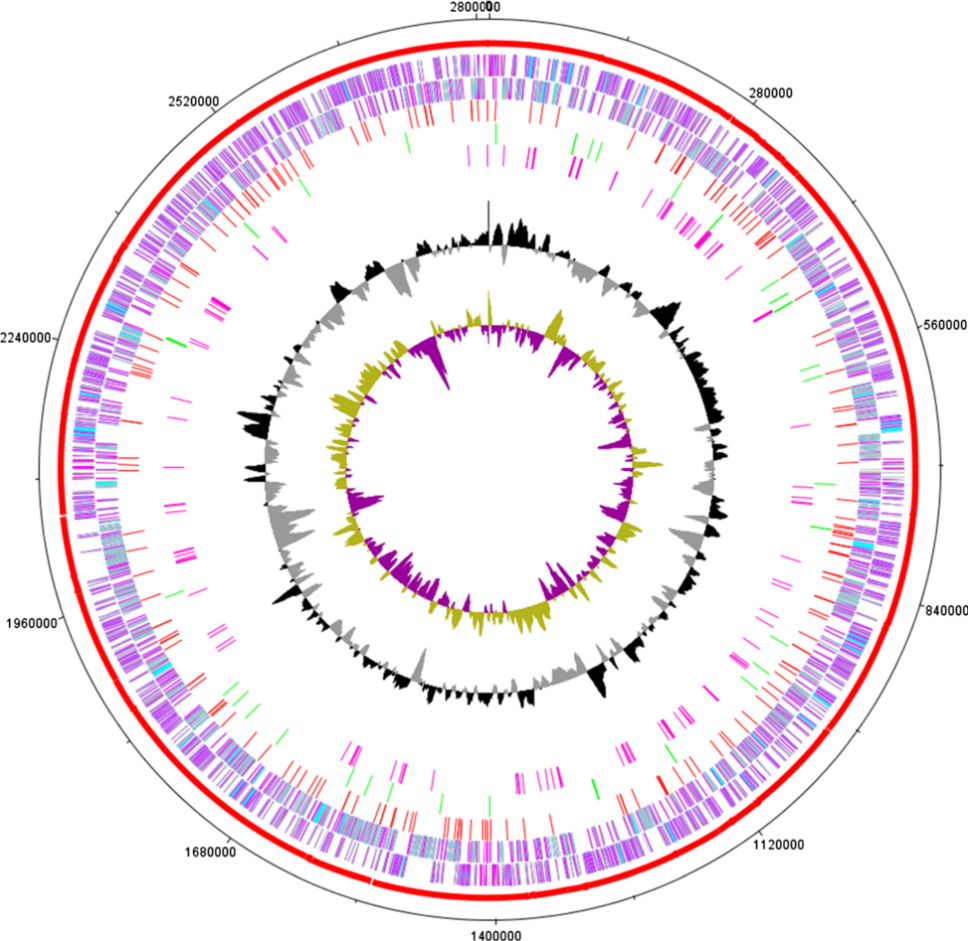
**Genome–atlas view of the** ***Prevotella intermedia*** **chromosome.** The outer to inner circles shows i)coordinates; ii) Contig + Singleton sequence; iii) positive CDS regions; iv)negative CDS regions; v) tandem repeats regions; vi) non-coding RNA regions; vii)CDD annotation; viii) GC content and ix)GC skew.

89 contigs of *Prevotella intermedia* ZT genome were assembled (Figure 1) while 91 singletons longer than 200 bp remained. 2165 ORF were predicted. The genome and annotations (BioProject ID: PRJNA208776; accession: ATMK00000000; BioSample SAMN02212661) have been submitted to NCBI.

### Genomic comparison of virulence factors in *Prevotella intermedia* strains

#### General features of genome comparison

We aligned the assembled contigs of *Prevotella intermedia* ZT with the published *P. intermedia* 17 genome (Accession: PRJNA163151; GenBank: CP003502.1 and CP003503.1) and *Prevotella intermedia* ATCC 25611 DSM 20706 (Accession: PRJNA185645; RefSeq: NZ_JAEZ00000000.1) using MUMmer3.23. One average, 88% of the genome is matched with the weighed identity of 96.2% between any two genomes.

**Figure 2:**
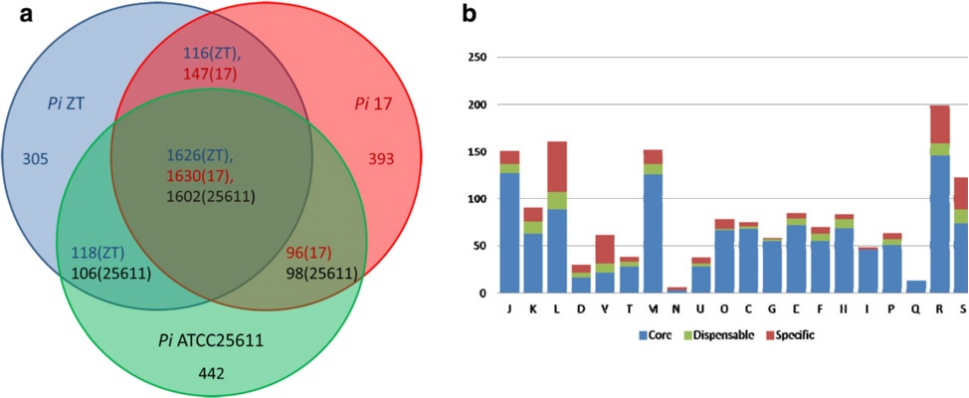
**Comparison of** ***Prevotella intermedia*** **ZT, 17 and ATCC 25611. a**, The Vann’s diagram of gene content difference of the three strains. **b**. the COG classification of the homologous gene clusters of *Prevotella intermedia* ZT, 17 and ATCC 25611. Core: Shared by all the three genomes; dispensable: Shared by part of the genomes; specific: strain specific. Abbreviation, function description and is shown as follows : D, Cell division and chromosome partitioning; M, Cell envelope biogenesis, outer membrane; N, Cell motility and secretion; V, Defense mechanisms; U, Intracellular trafficking and secretion; O, Posttranslational modification, protein turnover, chaperones; T, Signal transduction mechanisms; L, DNA replication, recombination, and repair; K, Transcription; J, Translation, ribosomal structure and biogenesis; E, Amino acid transport and metabolism; G, Carbohydrate transport and metabolism; H, Coenzyme metabolism; C, Energy production and conversion; P, Inorganic ion transport and metabolism; I, Lipid metabolism; F, Nucleotide transport and metabolism; Q, Secondary metabolites biosynthesis, transport, and catabolism; S, Function unknown; R, General function prediction only.

We clustered the genes of *Prevotella intermedia* ZT, 17 and ATCC 25611 and analyzed the COG classification. The numbers of core genes of the analyzed *Prevotella intermedia* strains are 1602, 1629 and 1602 (some clusters contains more than one genes from a genome) respectively, which is about 72% of all the genes encoded by each strain (Figure 2).

### Genes involved in adaptation and pathogenicity

The pathogenicity of anaerobic oral microbes usually initiates with the attachment and invasion of the host tissue. Pathogen will then break down certain cells or proteins of the host and later interfere with the immune system, stimulating inflammatory reaction [26]. Most of the genes involved in pathogenicity are shared by the three strains, but there are some strain-specific genes that contribute to adaption to niche and pathogenicity.

### Adhesion and attachment

Adhesion to the surface of the tooth or mucosa is the first step and an essential requirement for pathogen survival and pathogenicity in oral cavity or empyema. The structure of cell envelope is the foundation of adhesion to host tissue and surrounding microbes. 85% of the homologous gene clusters predicted to be involved in cell envelope construction are conserved among the three strains while there are strain specific genes: notably, *Prevotella intermedia* 17 has six strain-specific glycosyltransferase clustered together.

Besides that, some gene was reported to specifically contribute to adhesion. AdpB, an ompA family gene, has been reported to be related to adhesion to host tissue. AdpB, a 29 kDa protein, is the first broad-spectrum ECM (extracellular matrix)-binding protein localized on the cell surface that was identified and characterized in *Prevotella intermedia* 17 [24]. It shared by all the strains 6 orthologous clusters of adhesin are found in *Prevotella intermedia* strains but only one of them is shared by all the strains. Notably, *Prevotella intermedia* ZT has a tandem of 4 cleaved adhesin domain protein and 3 of them are strain-specific (Table 1).

**Table 1:** **The existence of adpB in the** ***Prevotella intermedia*** **strains and specific genes of cell envelope synthesis and adhesion**

***Pi*** **ZT**	***Pi*** **17**	***Pi*** **ATCC 25611**	**Annotation**
127001	387132175	2515940236	OmpA/adpB/Outer membrane protein and related peptidoglycan-associated (lipo)proteins
115010	-	-	Glycosyl transferase
105038	-	-	Energy transducer TonB
115011	-	-	Nucleotide sugar dehydrogenase
107096	-	-	Peptidase S41
-	387133452	-	Glycosyltransferase family protein
-	387133451	-	Glycosyltransferase, group 2 family protein
-	387133459	-	Glycosyltransferase, group 1 family protein
-	387133461	-	Sugar transferase
-	387133454	-	Glycosyltransferase, group 1 family protein
118017	387131650	2515939909	Cleaved adhesin domain.
115038	-	2515938020	Cleaved adhesin domain./CARDB.
-	387133158	2515938585	Cleaved adhesin domain./CARDB.
104072	-	-	Cleaved adhesin domain protein
104074	-	-	Cleaved adhesin domain protein
104075	-	-	Cleaved adhesin domain protein

The genes in the same raw belong to one homologous cluster with the threshold of e value 10^−10^, coverage 80% and identity 60%.

### Proteolysis and Bacteriocin

Protease or peptidase is one of the major virulence factors of *Prevotella intermedia*. Besides its role in degrading the host tissue, proteolysis is also an important part of the signaling pathway involved in various pathologies including inflammatory diseases [27,28]. Previous studies have shown that *Prevotella intermedia* degrades proteins such as immunoglobulin and LPS-binding protein (LBP) to impede the defense of the host [29-31].

**Figure 3:**
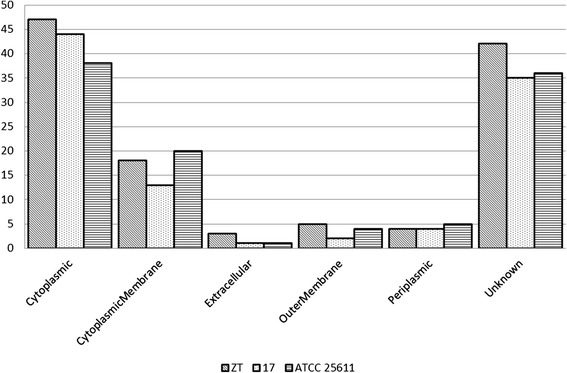
**The location of proteases encoded by** ***Prevotella intermedia*** **ZT, 17 and ATCC 25611.**

A large proportion of the proteases locate in cytoplasm and participate in the basic metabolism such as amino acid metabolism and posttranslational modification (Figure 3). Interestingly, Among the 24 strain specific proteases, 6 of them are bacteriocin or bacteriocin/lantibiotic exporters. The bacteriocins and lantibiotics also show great strain-specificity: except the two homologous cluster of lantibiotic ABC transporter and one cluster of bacteriocin, the rest of them are all strain specific. Bacteriocin and lantibiotic inhibit the grows of similar or closely bacterial strains. This indicate that the competition with the surrounding strains in the same niche, instead of breaking down proteins in the host tissues as we previously expected, may drive the evolution of *Prevotella intermedia* (Table 2).

**Table 2:** **Comparison of proteases and genes related with bacteriocin in the** ***Prevotella intermedia*** **strains**

**a. The specific proteases of the** ***Prevotella intermedia*** **strains. Note the genes involved in bacteriocin**			
***Pi*** **ZT**	***Pi*** **17**	***Pi*** **ATCC 25611**	**Annotation**
107096	-	-	Peptidase S41
108006	-	-	CAAX protease
108029	-	-	Hypothetical protein
114036	-	-	Peptidase M23
118026	-	-	DJ-1/PfpI family protein
126005	-	-	ATP-dependent serine protease
134012	-	-	Hypothetical protein
134014	-	-	Hypothetical protein
139007	-	-	Peptidase
142001	-	-	Bacteriocin
142003	-	-	Peptidase inhibitor I9 domain protein
142004	-	-	Bacteriocin
-	387131655	-	Putative bacteriocin
-	387131961	-	Putative bacteriocin
-	387132117	-	Putative caudovirus prohead protease
-	387132819	-	Putative caudovirus prohead protease
-	387133830	-	Hypothetical protein PIN17_A1967
-	387132710	-	Peptidase, M23 family
-	-	2515938070	ABC-type bacteriocin/lantibiotic exporters, contain an N-terminal double-glycine peptidase domain
-	-	2515938139	Hypothetical protein
-	-	2515938401	Hypothetical protein
-	-	2515939618	Membrane-bound metallopeptidase
-	-	2515939771	ABC-type bacteriocin/lantibiotic exporters, contain an N-terminal double-glycine peptidase domain
-	-	2515940199	Trypsin-like serine proteases, typically periplasmic, contain C-terminal PDZ domain
**b. The genes involved in bacteriocin in the** ***Prevotella intermedia*** **strains**			
103143	387132014	2515939098	Lantibiotic ABC transporter
120031; 140001; 141001	-	2515939774; 2515938065	ABC-type bacteriocin/lantibiotic exporters, contain an N-terminal double-glycine peptidase domain
-	-	2515938070	ABC-type bacteriocin/lantibiotic exporters, contain an N-terminal double-glycine peptidase domain
-	-	2515939771	ABC-type bacteriocin/lantibiotic exporters, contain an N-terminal double-glycine peptidase domain
120005	387131983; 387131657	2515939795; 2515939797	Bacteriocin/Subtilisin-like serine proteases
-	387131961	-	Putative bacteriocin
-	387131655	-	Putative bacteriocin
142001	-	-	Bacteriocin
142002	-	-	Bacteriocin
142004	-	-	Bacteriocin

### Iron uptake

Lysis of erythrocytes and degradation of hemoglobin are the major iron source of *Prevotella intermedia* [32]. This process begins with the accumulation of erythrocytes. According to a previous study, fimbriae of *Prevotella intermedia* induce hemagglutination [33], and the gene encoding hemagglutinin has been isolated [34,35]. Hemagglutinin, or mannosyl-glycoprotein endo-beta-N-acetylglucosaminidase, induces the aggregation of erythrocyte

*Prevotella intermedia* also has hemolytic activity [34,36-39]. When *Prevotella intermedia* is incubated in medium with rabit/sheep/humam red blood cells, beta-hemolytic zones are formed. The hemolytic activity reaches its maximum in weak acid condition [36]. The gene encoding hemolysin, the protein responsible for hemolytic activity, has been isolated and characterized [38]. Hemolysin gene is also identified in all *Prevotella intermedia* strains. Its homologous genes with the same/similar function are found in a wide range of *Prevotella* taxa

As to degredation of hemoglobin, *Prevotella intermedia* 17 encodes a cysteine protease named Interpain A (InpA), which breaks down oxyhaemoglobin by oxidating it to aquomethaemoglobin (in which the heam iron is oxidated to the Fe(III) state and has H_2_O as sixth co-ordinate ligand) at pH6.0. Aquemethaemoglobin is further completely broken down and hame is released to generate black-colored Fe(III) protoporphyrin IX pigment [40,41]. Interpain A also disturbs immune reaction of the host by degrading complement factor C3 [42]. The counterpart gene is identified in other *Prevotella intermedia* strains and its homologous proteins are found in *Prevotella nigrescens* and *Prevotella pallens*.

*Prevotella intermedia* strains share 2 copies of hemin-binding protein genes and a hemin receptor gene. Although PSORTb [43] fails to give any explicit prediction of the location of the two hemin-binding protein, their homologous genes in other *Prevotella spp.* are annotated to locate on the membrane. Hemin-binding protein identified in oral pathogen *Porphyromonas gingivalis* [44,45] and *Treponema denticola* [46] is a membrane protein that facilitates the uptake of hemin and the deletion of it will retard the growth of the bacteria in the iron-restricted media.

The whole pathway is intact in all the three strains. Although some genes with on the pathway are annotated to be hypothetic protein, they have more than 95% whole length identity with the genes annotated to be in the iron uptake pathway (Table 3).

**Table 3:** **The iron uptake pathway of** ***Prevotella strains***

***Pi*** **ZT**	***Pi*** **17**	***Pi*** **ATCC 25611**	**Annotation**
103136	387131911	2515939091	Hemagglutinin/mannosyl-glycoprotein endo-beta-N-acetylglucosaminidase/Muramidase (flagellum-specific)
103039	387131802	2515938961	Hemolysins/membrane protein, PF01595 family/Hemolysins and related proteins containing CBS domains
111047	387133496*	2515938788*	hemolysin/gliding motility-associated protein GldE
103117	387131889	2515939069*	Hemolysin secretion protein D/HlyD family secretion protein/Multidrug resistance efflux pump
109025	387132323*	2515939567	Hemolysin/acetyltransferase (GNAT) domain protein/Putative hemolysin
118018	387131649*	2515939908*	Interpain A/Peptidase C10 family.
107033	387132883*	2515939291*	Hemin-binding protein/hypothetical protein/Uncharacterized secreted protein
105015	387133319*	2515939710*	Hemin-binding protein/hypothetical protein/hypothetical protein
105006	387133309*	2515938428*	Hemin receptor/hypothetical protein/hypothetical protein
111047	387133496*	2515938788*	Hemolysin/gliding motility-associated protein GldE
103136	387131911	2515939091	Hemagglutinin/mannosyl-glycoprotein endo-beta-N-acetylglucosaminidase/Muramidase (flagellum-specific)
103039	387131802*	2515938961	Hemolysins/membrane protein, PF01595 family/Hemolysins and related proteins containing CBS domains
111047	387133496*	2515938788*	Hemolysin/gliding motility-associated protein GldE

The genes with annotation other than hemolysin and hemin-binding are marked with *.

### Drug resistance

*Prevotella intermedia* strains are predicted to encode different copies of beta-lactamases and multidrug/efflux transporters, which provide resistance to antibiotics. Genes of beta-lactamases and multidrug/efflux transporters are shared by almost all the three strains although in two of the homologous clusters, some of the genes are not annotated to be beta-lactamases despite of the more than 95% whole length identity with beta-lactamase gene (Table 4).

**Table 4:** **The genes involved in drug resistance**

***Pi*** **ZT**	***Pi*** **17**	***Pi*** **ATCC 25611**	**Annotation**
102115	387133861*	2515938326*	Beta-lactamase/BT1 family protein
103090	387131861	2515939024	Metallo-beta-lactamase/metallo-beta-lactamase domain-containing protein/Zn-dependent hydrolases, including glyoxylases
123014	387131920	2515940058	Beta-lactamase
107038	387132889	2515939286	Metallo-beta-lactamase
113052	387133548	2515938870	Metal-dependent hydrolases of the beta-lactamase superfamily III
107038	387132889	2515939286	Beta-lactamase class C and other penicillin binding proteins
105055	387133360	2515940206	Predicted Zn-dependent hydrolases of the beta-lactamase fold
103109	387131881	2515939060	Lipoate--protein ligase/Metal-dependent hydrolases of the beta-lactamase superfamily I
139005*	387132774	2515939613*	Flavoproteins/metallo-beta-lactamase domain-containing protein/Uncharacterized flavoproteins
146004	387132848	-	Beta-lactamase family protein
-	-	2515938733	Peptidase M23
-	-	2515938733	Predicted hydrolase (metallo-beta-lactamase superfamily)
101036	387132632	2515939689	ABC-type multidrug transport system, ATPase and permease components
101077	387132597	2515939351	ABC-type multidrug transport system, ATPase component
101098	387132587	2515939361	Predicted integral membrane protein
102063*	387133790	2515938274*	Cell division protein FtsX/efflux ABC transporter permease/Cell division protein
102069	387133795	2515938277	RND family efflux transporter, MFP subunit
102070	387133796	2515938278	Multidrug transporter AcrB/RND transporter, HAE1 family/The (Largely Gram-negative Bacterial) Hydrophobe/Amphiphile Efflux-1 (HAE1) Family
102071	387133797	2515938279	Efflux transporter, outer membrane factor (OMF) lipoprotein, NodT family
103067	387131831	2515938998	ABC-type multidrug transport system, ATPase component
103115	387131887	2515939067	ABC-type multidrug transport system, permease component
103116	387131888	2515939068	ABC-type multidrug transport system, permease component
103117*	387131889	2515939069	Hemolysin secretion protein D/Multidrug resistance efflux pump
104098	387133178	2515938563	Arabinose efflux permease
104115*	387133194	2515938543	GntR family transcriptional regulator/putative efflux protein, MATE family
106066	387132977	2515939203	ABC-type multidrug transport system, permease component
106067	387132976	2515939204	ABC-type multidrug transport system, permease component
106068	387132975	2515939205	ABC-type multidrug transport system, ATPase component
106069	387132974	2515939206	Multidrug resistance efflux pump
109065	387132263	2515940128	ABC-type multidrug transport system, ATPase and permease components
110015	387133713	2515938194	Putative efflux protein, MATE family
127020	387132192	2515938413	Putative efflux protein, MATE family
131014	387133631	2515938091	Putative efflux protein, MATE family
135009	387133260	2515938480	Putative efflux protein, MATE family
148002	387132059; 387133141	2515939982; 2515939845; 2515939937	ABC-type multidrug transport system, ATPase and permease components
103118*	387131890	2515939070*	Alkaline protease/outer membrane efflux protein/Outer membrane protein
119009*	387132415	2515940112	Membrane protein/efflux ABC transporter permease/ABC-type transport system, involved in lipoprotein release, permease component
101088	387133824	-	Multidrug DMT transporter permease
118030	387132859	-	MATE efflux family protein
-	387133140; 387132058	2515939938; 2515939981; 2515939846	ABC-type multidrug transport system, ATPase and permease components
-	387132973	2515939207	Outer membrane efflux protein/Outer membrane protein
-	387131718	-	Sugar efflux transporter for intercellular exchange
-	-	2515938842	Na + -driven multidrug efflux pump
-	-	2515939209	ABC-type multidrug transport system, ATPase component

The genes with annotation other than beta-lactamase and multidrug efflux transporter marked with *.

### Other feature related to virulence

Dipeptidyl peptidase IV(DPP4) are also found in the all the three *Prevotella intermedia* strains. DPP4 is a protease located on the surfaces of different cells across species including the genus *Prevotella* and also encoded by *Prevotella intermedia* 17 [47] and other 2 strains. Having a wide range of substrates [48], DPP4 is related to various physiological process including immune regulation, signal transduction and carcinogenesis [49-52]. A previous study showed that DPP4 from *Prevotella gingivalis* in gingival crevicular is associated with its pathogenicity and that *Prevotella* spp also has strong DPP activity [53]. This evidence points to an important role for DPP4 in pathogenicity.

Hypothetical virulence factor BrkB (103055), a 448-amino acid protein, was also found shared by the three strains. Virulence factor BrkB was first identified in *Bordetella pertussis* and was found to be responsible for serum resistance [54]. It is also shared by the three strains.

Virulence-associated protein E and other toxin proteins were identified in the three strains. The strains both share several copies of homologous genes and each possesses strain-specific genes (Table 5).

**Table 5:** **Other genes involved in drug resistance**

***Pi*** **ZT**	***Pi*** **17**	***Pi*** **ATCC 25611**	**Annotation**
132012	387133613	2515938073	Peptidase S9/Dipeptidyl aminopeptidases/acylaminoacyl-peptidases
103055	387131819	2515938987*	Hypothetical virulence factor BrkB/ Predicted membrane protein
118023; 149001; 116050; 101089	387132842; 387133147; 387133822	2515938146; 2515939042	Virulence-associated protein E/Virulence-associated protein E/Predicted P-loop ATPase and inactivated derivatives
122010	387132071	2515939824	Toxin HipA/HipA-like C-terminal domain./HipA-like N-terminal domain
138006	-	2515940043	Toxin Fic/Virulence protein
106093	-	-	Virulence-associated protein E
-	387132952	-	Virulence-associated protein E
-	-	2515939988	Virulence-associated protein E.
-	-	2515939916	Virulence protein
-	387131936	-	Toxin-antitoxin system, toxin component, Fic domain protein
-	-	2515939808	Addiction module toxin, RelE/StbE family
-	387131936	-	Toxin-antitoxin system, toxin component, Fic domain protein

The genes with annotation other than beta-lactamase and multidrug efflux transporter marked with *.

### Recombination in *Prevotella intermedia*

There are more about 130 genes predicted to have functions of replication, recombination and repair in each *Prevotella intermedia* genome. Recombinase, integrase and type I restriction enzymes and clusters of conjugal transfer protein genes are found in the genomes (data not shown). We also found cluster of strain-specific genes (Additional file 1) in all three strains, which are possibly the relic of previous transpositions.

Some of these clustered strain-specific genes are potentially related with adaptation and pathogenicity: *Prevotella intermedia* ZT has a cluster containing 1,3-beta-glucan synthase regulator genes; *Prevotella intermedia* 17 has a cluster of ABC transporter genes and a cluster of glycosyltransferase genes; *Prevotella intermedia* ATCC 25611 has a cluster containing glycosyltransferases and Na + -driven multidrug efflux pump and a cluster containing BC-type bacteriocin.

### Genomic comparison of gene content within the genus *Prevotella*

In addition to the intro-species genome alignment between *Prevotella intermedia* ZT and *Prevotella intermedia* 17, we aligned the *Prevotella intermedia* ZT genome against the genomes of *Prevotella dantalis* DSM 3688 (NCBI ID: 184818), *Prevotella melaninogenica* ATCC 25845 (NCBI ID: 51377), *Prevotella denticola* F0289 (NCBI ID: 65091), *Prevotella ruminicola* 23 (NCBI ID: 47507) (Additional file 2). The alignments show that the genome sequences are barely matched between two different species. The matched region count only ~3% of the whole genome in the nucleotide sequence. However a ~49Kb region that is conserved in terms of gene content and partly DNA sequence is shared by all the considered genomes (Table 6). This region located around from 56 Kb to 95 Kb of contig00007 in the draft *Prevotella intermedia* ZT genome sequence. It encodes DNA-directed RNA polymer, 30S and 50S ribosomal proteins and translation elongation factor and five tRNA. And the corresponding regions in other genomes have similar gene contents and arrangement.

**Table 6:** **The conserved region across different taxa**

**Strains**	**Contig or Chromosome**	**Range**
*P. intermedia* ZT	contigs00007	56Kb to 95Kb
*P. intermedia* 17	gi|387132089|ref|NC_017861.1|	986Kb to 1024 kb
*P. dentalis*	gi|433651058|ref|NC_019960.1|	274 kb to 326 kb
*P. denticola*	gi|327312315|ref|NC_015311.1|	1066Kb to 1110Kb
*P. melaninogenica*	gi|302344773|ref|NC_014370.1|	412b to 457 kb
*P. ruminicola*	gi|294672793|ref|NC_014033.1|	2570Kb to 2522Kb

### COG analysis

In addition to the Cluster of Orthologous Group (COG) [55] distribution of *Prevotella intermedia* ZT, the Pan-genome of *Prevotella intermedia* ZT, *Prevotella intermedia* 17 (NCBI ID: 163151), *Prevotella dantalis* DSM 3688 (NCBI ID: 184818), *Prevotella melaninogenica* ATCC 25845 (NCBI ID: 51377), *Prevotella denticola* F0289 (NCBI ID: 65091), *Prevotella ruminicola* 23 (NCBI ID:47507) was also annotated.

Unlike the COG analysis of a single genome, COG analysis of pan-genome gives the number of gene clusters classified with certain annotation instead of the number of genes. The COG result is stratified based on the gene cluster level given by PGAP1.1. The gene cluster level is the number of the genome that distribute to a certain cluster. If a cluster has the cluster level of *n*, it has the genes from *n* genome(s). Core clusters consist of genes from all the analyzed genomes (in our study, they have the cluster level of 6). Strain-specific clusters consist of genes from only one genome and have the cluster level of 1. The rest are dispensable clusters. Whole-cluster presents the gene function distribution of all the genes in the pan-genome, namely, the sum of core, dispensable and strain-specific clusters.

**Figure 4:**
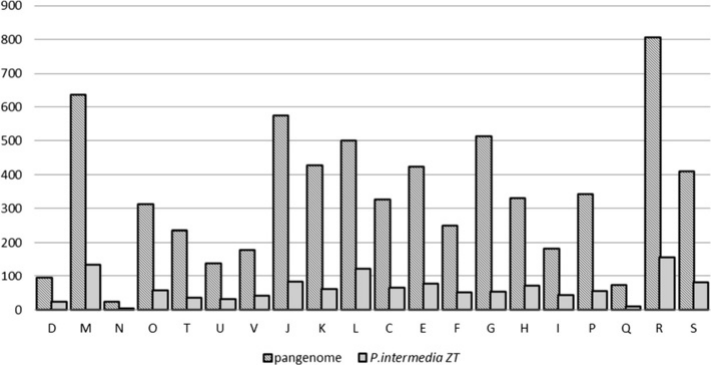
**The COG function classification of** ***Prevotella intermedia*** **ZT’s genes and** ***Prevotella*** **pan-genome gene clusters.**

As expected, *Prevotella intermedia* ZT has a similar COG distribution with the whole-cluster of pan-genome (Figure 4). The function class with relatively high number of gene clusters in the pan-genome also has relatively high number of genes in *Prevotella intermedia* ZT genome and *verse visa*. As taxa of the same species, they reasonably share some common features.

**Figure 5:**
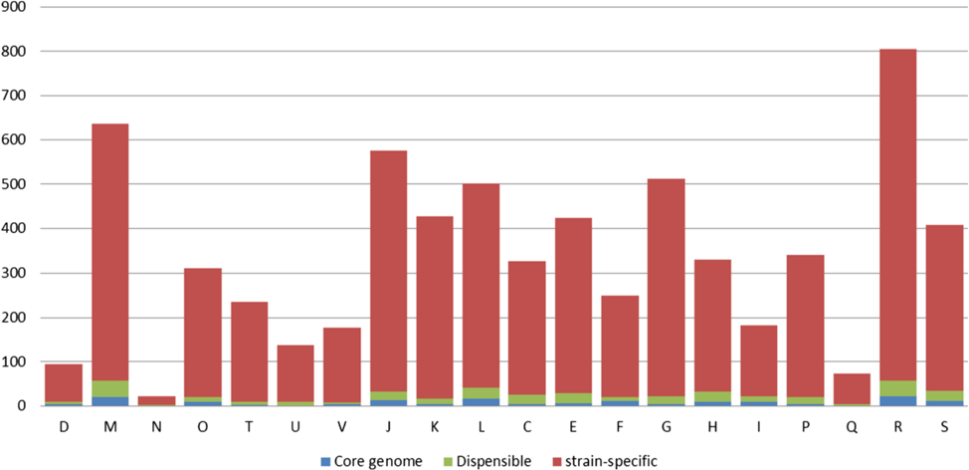
**The COG classification of the homologous gene clusters of** ***Prevotella*** **pan-genome.** Note that for every COG class, the strain-specific clusters are the majority.

The more striking feature of the analyzed taxa is the specific genes. For every COG class, the major of the clusters are strain-specific (Figure 5) which accords with the results drown from the mathematics model that the genus *Prevotella* has a considerable number of strain-specific genes.

The genes classified into the “intracellular trafficking, secretion and vesicular transport” is absent in the core genome but enriched in strain-specific cluster. The high species specificity of secretion function in the genus *Prevotella* indicates the high specificity of the interaction between *Prevotella* taxa and its niche.

There is no gene in “cell motility” class in the core genome either. However, considering the fact that “cell motility” has the least genes in every single genome and that KEGG pathway analysis shows that the genus *Prevotella* generally has no or only one gene in flagella synthesis pathway, the absence of “cell motility” gene in the core genome should be due to the low motility and probably the absence of flagella of the genus *Prevotella*. We assume that the genus *Prevotella* make use of other structures or the help of the surrounding microbe populations to attach on the surface of the oral cavity and invade into the abscess tissue.

### Pan-genome analysis and gene distribution mathematics model

Pan-genome is originally used to describe the core genome of all taxa of a species typically the bacteria and archaea species [56]. Considering the similarity between different species of the genus *Prevotella*, the term pan-genome is used to describe the essential genes of the genus *Prevotella*. For the sake of data validity we use only the completely sequenced *Prevotella* taxa and *Prevotella intermedia* ZT to perform the pan-genome analysis. The completely sequenced genomes are *Prevotella intermedia* 17 (NCBI ID: 163151), *Prevotella dantalis* DSM 3688 (NCBI ID: 184818), *Prevotella melaninogenica* ATCC 25845 (NCBI ID: 51377), *Prevotella denticola* F0289 (NCBI ID: 65091), *Prevotella ruminicola* 23 (NCBI ID: 47507).

The genes from the six genomes were clustered with PGAP1.1 and only bidirectional best hits were further analyzed. The genus *Prevotella* has about 165-170 core genes, that is, the genes shared by all the analyzed taxa. This number slightly varies between different genomes because of gene duplications or homology. Each genome has strain-specific genes ranging from 1418 to 2763 (Table 7).

**Table 7:** **The gene conservation distribution of each genome in the pan-genome analysis**

**Shared by taxa**	**6**	**5**	**4**	**3**	**2**	**1**	**Total**
*Prevotella intermedia* ZT	165	146	123	59	135	1439	2067
*Prevotella intermedia* 17	166	142	105	68	117	1668	2266
*Prevotella dentalis* DSM 3688	167	31	7	7	4	1202	1418
*Prevotella melaninogenica* ATCC 25845	169	143	96	25	4	1859	2296
*Prevotella denticola* F0289	169	142	102	20	0	1953	2386
*Prevotella ruminicola* 23	170	119	11	13	1	2449	2763

Venn diagram is usually used to show the distribution of conserved and strain-specific genes. However it will be too fuzzy when more than three genomes are to be considered. Therefore, we take advantage of the mathematics model provided by the PGAP to explicit the gene distribution of the genus *Prevotella*.

The mathematics model exhausts the pan-genome and core genome size generated from every possible combination of genomes and fit the data with a power model for pan-genome and an exponent model for core-genome:

$$ \begin{array}{l}y=a{x}^b+c\left(a,\kern0.5em b,\kern0.5em c>0\right)\hfill \\ {}z=m{e}^{-nx}+k\left(m,\kern0.5em n,\kern0.5em k>0\right)\hfill \end{array} $$

where *y* denotes the number of genes in the pan-genome; *z* denotes the number of genes in the core genome; *x* denotes the genome number and *a, b, c, m, n, k* are the fitting parameter.

In the pan-genome model, parameter *b* is less than 1 because the contribution of each extra single genome added to the pan-genome analysis tends decrease due to the homologous genes between the each genome. From the model we can calculate some important parameter: *y*(1) is the average gene number of a single genome; *y*(2)- *y*(1) is the average difference in gene number of the pan-genome constructed from any two genome and a single genome, that is, the average strain-specific genes in a genome when any two genomes are compared.

In the core genome model, the index of *e* is minus because the size of the core genome decreases when more genomes are included in the pan-genome analysis. Similarly, *z*(1) is also the average gene number of a single genome and *z*(1)-*z*(2) is the average strain-specific genes in a genome when any two genomes are compared.

The mathematics model based on the six previous mentioned genomes is:

$$ \begin{array}{l}y=1994.3{x}^{0.96}+23.3\hfill \\ {}z=32442.4{\mathrm{e}}^{\hbox{-} 2.8}x+202.8\hfill \end{array} $$

In this model, *y*(2)- *y*(1) is 1838.0, which is more than half of the genes in a *Prevotella* genome. This mathematics model shows that on average more than half of the genes are strain-specific between any two species.

We expand the sample size and build the mathematics from 29 species of the genus *Prevotella* (either completely sequenced genomes or draft genomes. For each species we only chose one genome in order to prevent that the intra-species differences will not interfere the model) and draw a similar conclusion. In the new model, the average genome size is slightly larger than the previous one and the number of strain-specific genes in a single genome is notably lowered. Even so, the number of strain-specific genes between any two genomes is a considerable 1232, which is nearly half of the genes encoded by a genome.

$$ \begin{array}{l}y=1938.9{x}^{0.71}+505.9\hfill \\ {}z=3241.2{\mathrm{e}}^{\hbox{-} 0.62\mathrm{x}}+533.5\hfill \end{array} $$

### Phylogenetics of the genus *Prevotella*

**Figure 6:**
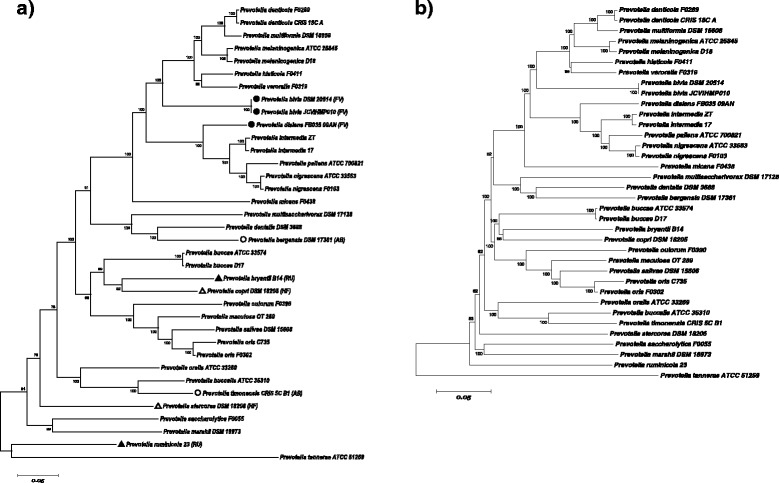
**The phylogenetic tree of the genus** ***Prevotella*** **. a** The phylogenetic tree build by Maximun-Likelihood Method. The isolation sites are marked on the phylogenetic tree. unmarked: oral cavity; △: human feces (HF);▲: rumen(RU); ○: abscesses (AB);●: vagina (FV). **b**. The phylogenetic tree build by Neighbor-joining Method.

The phylogenetic tree is a common methodology to infer the evolutionary relationship between different species. We selected among the genes that the 36 *Prevotella* taxa have in common (Additional file 3) and got 80 sets of homologous genes (Additional file 4). These genes are involved in carbohydrate utilization, cell component construction, genetic information process and other basic biochemistry reactions. The 80 sets of genes were used to generate a set of concatenated 34581-nt sequences to build the phylogenetic tree. Maximum likelihood and neighbor joining method were used and as expected generated almost identical results which shared the very same topology and strains of the same species were closest to each other (Figure 6).

In order to reveal the influences of niche on the evolution of core genes of the genus *Prevotella*, we referred to the NCBI websites and previous literature for the isolation sites of each taxon and combined the isolation sites with the phylogenetic tree. Despite the possibility that the bacteria may survive and thrive in the niches other than the isolation sites, the isolation sites at least indicate the niches which the bacteria most successfully adapt to. The majority of the analyzed taxa were isolated from oral cavity but a few taxa were isolated from vagina (*Prevotella bivia, Prevotella disiens*), human feces (*Prevotella stercorea, Prevotella copri*), rumen and (*Prevotella ruminicola, Prevotella bryantii*) and abscesses (*Prevotella bergensis, Prevotella timonensis*). These exceptional species are scattered over different branches on the phylogenetic tree, which suggests that adapting to different niches did not play an essential role in the early evolutionary history of the genus *Prevotella* but accelerated the speciation in the late history.

The phylogenetic tree shows that the *Prevotella intermedia* has a close evolutionary relationship with *Prevotella pallens*, *Prevotella nigrescens* and *Prevotella disiens*. The former two species are usually isolated from oral cavity while the latter, *Prevotella disiens* is a pathogen that mainly exists in vagina [57,58].

### Amino acid metabolic pathway analysis

As a pathogen which has already adapted to protein-rich niches, *Prevotella intermedia* ZT lacks many biosynthesis pathways, especially those which synthesize small-molecule nutrients such as amino acid.

The pathways of amino acid metabolism and biosynthesis are very limited. According to the KEGG pathway analysis [59], the strain *Prevotella intermedia* ZT does not have biosynthesis pathway for most of amino acids with only two exceptions: the synthesis of glutamine and glutamate, which plays an important role in lipopolysaccharide biosynthesis, and the biosyntheses of serine from hydroxylpyruvate. Besides that, the strain *Prevotella intermedia* ZT possesses some enzymes that catalyze the synthesis of amino acid from other amino acid and immediate metabolite: 1) the glycine hydroxymethyltransferase that transform glycine to serine and also the reverse reaction, 2) saccharopine dehydrogenase that degrades saccharopine into lysine, 3) L-asparaginase that transforms asparagines to aspartic acid and 4) branched-chain amino acid aminotransferase that catalyzes the reversible transformation of valine, leucine and isoleucine to their immediate metabolites 2-oxoisovalerate, 4-methyl-2-oxopentanoate and (S)-2,3-methyl-2-oxopentanoate. The previous sequenced strain *Prevotella intermedia* 17 shares all the amino acid metabolism pathway features with *Prevotella intermedia* ZT with some minor differences.

The analysis of other *Prevotella* taxa, that is, *Prevotella dantalis* DSM 3688 (NCBI ID: 184818), *Prevotella melaninogenica* ATCC 25845 (NCBI ID: 51377), *Prevotella denticola* F0289 (NCBI ID: 65091), *Prevotella ruminicola* 23 (NCBI ID: 47507) shows that the genus *Prevotella* vary greatly in terms of amino acid metabolism. *Prevotella ruminicola* has the ability to generate every amino acid from other metabolites, which probably results from the carbonhydrate-rich but peptide-deficient ruminal environment. Other taxa more or less have lost the genes involved in amino acid biosynthesis and the loss of genes is most severe in *Prevotella intermedia*.

In terms of histidine metabolism, *Prevotella intermedia* has lost every gene in the histidine biosynthesis pathway but possesses a pathway to degrade histidine. *Prevotella denticola* and *Prevotella melaninogenica* have the same histidine degradation pathway as *Prevotella intermedia* does and they also lost most of the gene in the hisitidine biosynthesis pathway except histidinol-phosphate aminotransferase which generate L-histidinol phosphate with the translocation of an amino group from Imidazole-acetol phosphate to L-glutamate. In sharp contrast with *Prevotella intermedia, Prevotella ruminicola* has an intact histidine biosynthesis pathway but does not encode any enzymes to degrade hisitdine. Interestingly, *Prevotella dentalis* is similar with *Prevotella ruminicola* although it is isolated in oral cavity. It has no enzymes to metabolize histidine but a nearly full biosynthesis pathway with only two genes lost.

As to valine, leucine and isoleucine biosynthesis pathway, *Prevotella intermedia, Prevotella denticola and Prevotella melaninogenica* have lost all the genes except the branched-chain amino acid aminotransferase which also locates in valine, leucine and isoleucine degradation pathway. On the contrary, *Prevotella ruminicola* and *Prevotella dentalis* have a full biosynthesis pathway.

As to the synthesis of phenylalanine and tyrosine, all the analyzed taxa have some genes lost and the pathway in every genome is incomplete. The pathway can be divided into two parts: the biosynthesis of intermediate metabolite prephenate and the synthesis of phenylalanine and tyrosine from prephenate. All the taxa encode some of the enzymes involved in the biosynthesis of chorismate, the immediate metabolite that converts to prephenate reversibly. But they do not encode chorismate mutase, the enzyme that catalyzes this conversion. Both of the *Prevotella intermedia* strains have lost all the genes for synthesizing phenylalanine and tyrosine from prephenate while other taxa maintain two (*Prevotella melaninogenica*) or four genes (*Prevotella dentalis, Prevotella denticola, Prevotella ruminicola*).

The summary of the ability to convert other metabolite to certain amino acid is given in the Table 8. Expectedly, some conclusion about the amino acid metabolism of *Prevotella intermedia* drawn from the genome analysis accords with a previous bench experiment [60].

**Table 8:** **The ability to convert other metabolites to amino acid**

**Amino Acid**	***P. intermedia*** **ZT**	***P. intermedia*** **17**	***P. dentalis***	***P. denticola***	***P. melaninogenica***	***P.ruminicola***
Alanine	no	no	no	no	no	yes
Arginine	no	no	yes	no	no	yes
Asparagine	no	no	yes	yes	yes	yes
Aspartic acid	yes	yes	no	yes	yes	yes
Cycteine	no	no	yes	yes	yes	yes
Glutamate	yes	yes	yes	yes	yes	yes
Glutamine	yes	yes	yes	yes	yes	yes
Glycine	yes	yes	yes	yes	yes	yes
Histidine	no	no	yes	no	no	yes
Isoleucine	no	no	yes	no	no	yes
Leucine	no	no	yes	no	no	yes
Lysine	yes	yes	yes	yes	yes	yes
Methionine	no	no	yes	no	yes	yes
Phenylalanine	no	no	yes	yes	yes	yes
Proline	no	no	no	no	no	yes
Serine	yes	yes	yes	yes	no	yes
Threonine	no	no	no	no	no	yes
Tryptophan	no	no	yes	yes	yes	yes
Tyrosine	no	no	yes	yes	yes	yes
Valine	no	no	yes	no	no	yes

The data is predicted from the KEGG pathway analysis and not yet confirmed experimentally.

The considerable differences in amino acid biosynthesis indicate the readiness of evolution under proper environment. The ability to synthesize amino acids, which are quite available from the host tissue, is in the process of degradation.

### Carbohydrate metabolic pathway and energy production

As an anaerobic microbe, the genus *Prevotella* only possesses a limited repertoire of enzymes for carbohydrate metabolism and energy production. The enzyme content varies from species to species. *Prevotella intermedia* ZT and *Prevotella intermedia* 17 have relatively incomplete pathways but the enzyme repertoire in *Prevotella dentalis* is the most reduced. Compared with the oral-isolated taxa, the rumen isolated *Prevotella ruminicola* has the most comprehensive repertoire of carbohydrate usage enzyme (Table 9).

**Table 9:** **The number of genes in main pathways of carbohydrate metabolism and energy product**

**Taxa**	**Glycolysis/Gluconeogenesis**	**Starch and sucrose metabolism**	**Pyruvate metabolism**
*P. intermedia* ZT	17	16	11
*P. intermedia* 17	17	16	12
*P. dentalis*	10	13	8
*P. denticola*	19	22	14
*P. melaninogenica*	20	20	15
*P. ruminicola*	20	24	12

Glycolysis is the first step to metabolize glucose and energy production for every species. In our study, almost all the taxa that we analyze have a functional glycolysis pathway except *Prevotella dentalis*, which lacks glucose-6-phosphate isomerase and 6-phosphofructokinase 1. These two enzymes catalyze the second and third reaction (transform α-D-glucose-6P to β-D-Frucose-1,6P_2_) of the glycolysis. *Prevotella dentalis* also lacks glyceraldehyde 3-phosphate dehydrogenase, the enzyme that catalyze the fifth reaction that transform D-glyceraldehyde 3-phosphate to 3-phospho-D-glyceroyl phosphate and several enzymes in the flanking branches of the glycolysis pathway.

In addition to glycolysis, we analyze other enzymes involved in carbohydrate metabolism. According to the KEGG pathway mapping, the genus *Prevotella* do not produce lactate or ethanol in cellular respiration due to the absence of the relative enzymes in the glycolysis pathway. Instead, in the pyruvate metabolism pathway, the pyruvate generated during glycolysis are the raw material for the synthesis of formate, oxaloacetate, which accords with the previous research [61]. These metabolites, especially noxious ones such as formate may contribute to the pathogenicity of the genus *Prevotella*.

### Synthesis of cell wall components

Considering the analyzed taxa, the genus Prevotella has relatively complete, functionally conserved pathways for the synthesis of complex molecules that are used for cell material such as lipopolysaccharide and peptidoglycan.

Components such as lipopolysaccharides (LPS) [62-70] induce immunological reaction of the host. In the *Prevoeolla intermedia* ZT genome, we identified genes related to lipopolysaccharides synthesis. The enzymes encoded by these genes compose a nearly full pathway from primal raw material UDP-N-acetyl-D-glucosamine to KDO_2_-lipid IV(A) (KEGG ID: C06025) except that the 3-deoxy-D-manno-octulosonate 8-phosphate phosphatase (KDO 8-P phosphatase) is missing. This enzyme catalyzes the transformation from 3-deoxy-D-manno-octulosonae-8P to 3-deoxy-D-manno-octulosonate. Considering the economy of microbe metabolism, we conjectured that it was not likely that the whole biosynthesis of LPS should be interrupted by one single missing gene and therefore there might be other mechanism that hydrolyzed the phosphate group of 3-deoxy-D-manno-octulosonae-8P. Besides that, no enzyme on other branches of LPS synthesis has been found in this study. From the facts mentioned above, it is natural to draw the conclusion that the main component of LPS of *Prevotella intermedia* ZT is KDO_2_-lipid IV(A).

**Figure 7:**
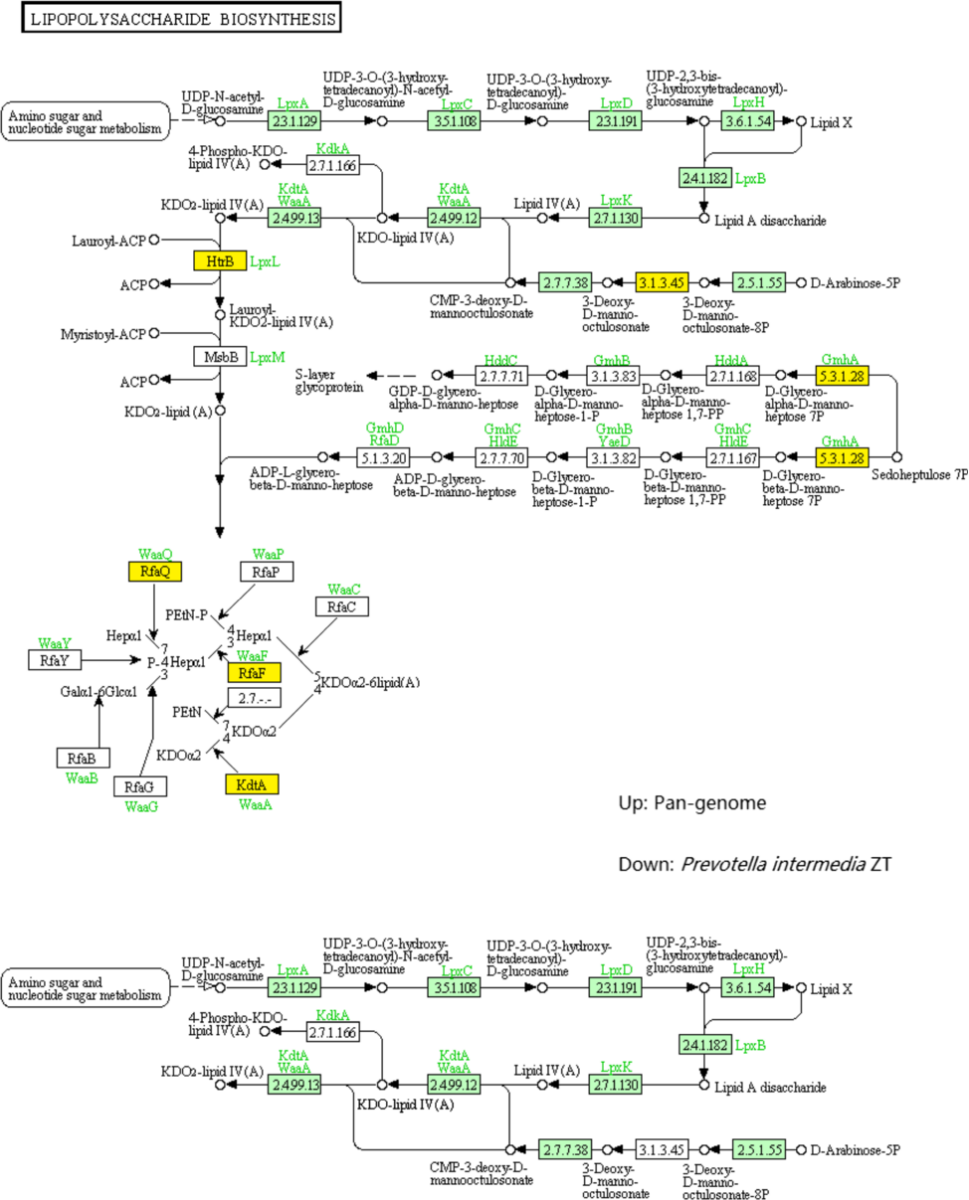
**Up: The pan-genome LPS biosynthesis pathway.** The core genes are marked with green and the dispensable/specific genes with yellow. Down: The *Prevotella intermedia* ZT LPS biosynthesis pathway. The uncolored genes do not exist in the genome; They are shown in the graph to make the pathway explicit and integrated.

Other taxa have a similar LPS biosynthesis pathway. Generally, the *Prevotella* has a functional pathway to synthesize KDO_2_-lipid IV(A) but a few of enzymes in the branch pathway to modify the final product vary with taxa (Figure 7).

**Figure 8:**
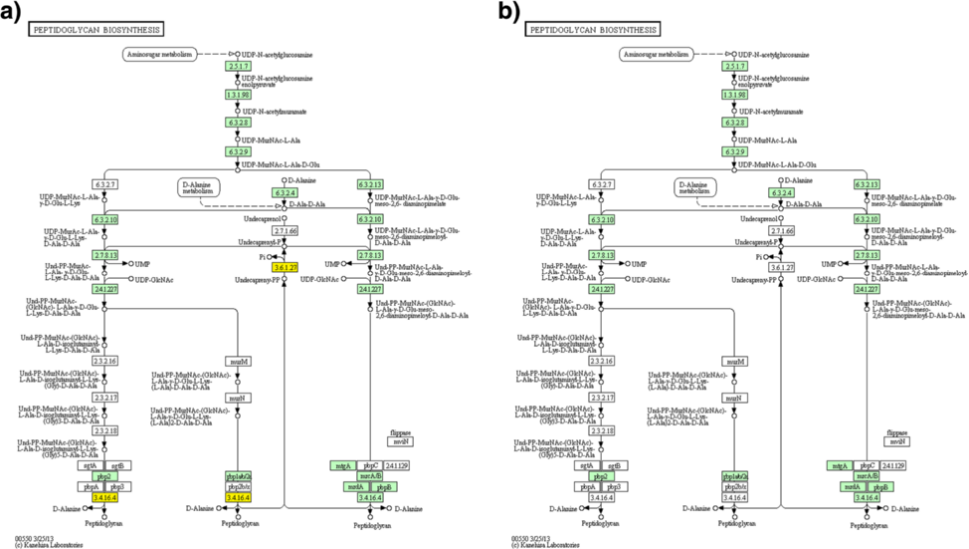
**Peptidoglycan biosynthesis pathway in** ***Prevotella intermedia*** **ZT and the pan-genome of the genus** ***Prevotella*** **. a**. The pan-genome peptidoglycan biosynthesis pathway. The core genes are marked with green and the dispensable/specific genes with yellow. **b**. The *Prevotella intermedia* ZT peptidoglycan biosynthesis pathway. The uncolored genes do not exist in the genome; They are shown in the graph to make the pathway explicit and integrated.

The synthesis of another important bacteria cell wall component peptidoglycan is also well equipped with a fully functional pathway (Figure 8). Compared with *Prevotella intermedia* 17, *Prevotella intermedia* ZT lacks only one gene, undecaprenyl-diphosphatase which hydrolyzes a phosphate from ditrans,octacis-undecaprenyl diphosphate. Although there are some disconnected branches in the peptidoglycan pathway, the main string of the pathway is unblocked. Besides, the blocked branches on the biosynthesis pathway indicate a corresponding biased peptidoglycan composition: The most likely major protein components of the peptidoglycan of genus *Prevotella* is undecaprenyl-diphospho-N-acetylmuramoyl-(N-acetylglucosamine)-L-alanyl-D-glutamyl-meso-2,6-diaminopimeloyl-D-alanyl-D-alanine (KEGG entry: C05898) and undecaprenyl-diphospho-N-acetylmuramoyl-(N-acetylglucosamine)-L-alanyl-gamma-D-glutamyl-L-lysyl-D-alanyl-D-alanine(KEGG entry: C05893).

## Conclusions

As an important pathogen, *Prevotella intermedia* has long interested stomatologists and microbiologists. It is of clinical significance to study what is the potential drive of its adaptation and pathogenicity in different niches. In previous study, *Prevotella intermedia* 17, a clinically isolated strain from a human periodontal pocket and *Prevotella intermedia* ATCC 25611, the type strain isolated from empyema were sequenced. We isolated the strain *Prevotella intermedia* ZT from the infected root canal of a Chinese patient with chronic apical abscess and compared the genome content of this strain with other *Prevotella* taxa.

Our study confirmed that *Prevotella intermedia* ZT possessed a range of virulence factors involved in adhesion, proteolysis, iron uptake etc. For every aspect of pathogenicity, the involved genes have a relatively conserved part and a strain-specific part. The level of conservation varies with the aspect of pathogenicity, which indicates that the different strains of *Prevotella intermedia* inherited a basic package of genes enabling it to adapt to different niches and cause diseases, and later the package diverged under different conditions and the genes of different functions evolved at different rates. As far as this study shows, strain specificity is greater within genes involves in adhesion and the product and secretion of bacteriocin. It shows that, instead of exploiting the resources from the host, the most important variables of adaptation and pathogenicity are adhesion to the host tissue and/or surrounding microbes and competing with surrounding microbes. Besides, the activity of recombination shows that *Prevotella intermedia* may gain pathogenic genes through horizontal gene transfer.

Considering the similarity of the living environment and basis gene contents of the genus *Prevotella*, we analyze other *Prevotella* taxa to get a better view of what aspect of adaptation and pathogenicity matters most for the survival of *Prevotella*.

Our study shows that *Prevotella* taxa have very dynamic genomes with a part of relative high conserved contents. While comparing different taxa across species, we also find the existence of a conserved part. The genus *Prevotella* has a set of core genes involved in almost every COG class except “intracellular trafficking, secretion and vesicular transport” and “cell motility”. The genomes also share an ~49 kb conserved region involved in ribosome biosynthesis and translation. The pathways involved in carbohydrate metabolism and cell wall component synthesis are relatively conserved among the genus *Prevotella*. The anaerobic respiration product of genus *Prevotella* is neither lactate nor ethanol but potentially noxious chemicals such as formate. On the other hand, *Prevotella* taxa have very dynamic genomes. From the mathematics model, we draw the conclusion that the genus *Prevotella* have highly dynamic genomes that vary greatly in sequences and gene contents from species to species: On average any two genomes of different species share only half of the genes. The strain-specific genes make a large part of each COG cluster in the pan genome, which allows the taxa of the genus *Prevotella* to adapt to various niches and to show various pathogenicities. The genus *Prevotella* display great difference in amino acid biosynthesis. Rumen-isolated species *Prevotella ruminicola* maintains the ability to synthesize all the amino acid while other oral taxa more or less lost some genes to convert other metabolites to amino acids. The gene loss in *Prevotella intermedia* is most severe.

This result shows that *Prevotella* taxa which parasitize in oral cavity have enough nitrogen sources so that they gradually lost gene of amino acid synthesis. However, the function of cell wall synthesis remains intact under a highly dynamic context, which accords with our previous hypothesis that adhesion is one of the main drives of the evolution of *Prevotella intermedia*.

With a highly dynamic genome which enables fast adaptation, metabolic pathway that product potentially noxious products and a wide range of virulence factors, *Prevotella intermedia* are well equipped for survival in different niches and pathogenicity. Our study shows that instead of exploiting the resources from the host, adhesion and the competition and horizontal gene transfer with the surrounding microbes may be the main drives of the evolution of *Prevotella intermedia*.

However, since the core content of the *Prevotella intermedia* has already provide the basic function for each process of pathogenicity, the variation in pathogenic genes is not very significant. Actually it leaves a question whether the variation causes or is caused by adaptation to different niches. Limited by the data resources, we cannot fully answer and validate this question, but we assumed that the almost full function of core content can explain why *Prevotella intermedia* is able to survive in various niches.

We use a new method to evaluate the variation of genome content. Venn diagram is a common method to visualize the differences in the gene contents. It is explicit and easy to understand but troublesome when more than 5 genomes are analyzed due to the limited expression of flat graph. Here we make use of a mathematics model provided by PGAP to summarize and quantify the tendency of the gene content variation between different genomes. The mathematics model can be served as a supplementary for Venn diagram.

## Method

### The collection of clinical samples

#### Selection of patient

The sample was collected from a primary periapical periodontitis patient (male, 34 years old) who had sought treatment for endodontic treatment of his mandibular right canine (tooth 43) diagnosed with apical periodontitis. Biting pain, percussion pain and tenderness to palpation were presented. There was a sinus tract over the buccal mucosa of tooth 44. Gutta-percha tracing of the sinus tract indicated the lesion was originated from the apical region of tooth 43.

The patients had no systematic diseases and had received no previous pulp and periodontal therapy and no antibiotics usage within 3 months before sample collection. The tooth harboring pulpal necrosis was sampled and the tooth was intact enough to allow adequate isolation from saliva.

We obtained the written informed consent from the patient, and the study was approved by the Ethic Committee of Shanghai 9th People’s Hospital affiliated to Shanghai Jiao Tong University, School of Medicine, China. The age, gender, general conditions, prior oral treatment and preoperative radiograph were recorded.

### Sample collection

The microbiological samples were collected from the infected root canals as described in previous studies [16,71]. After isolated from the oral cavity by a disinfected rubber dam, the tooth was disinfected with 3% hydrogen peroxide followed by 2.5% sodium hypochlorite, which was inactivated with 5% sodium thiosulfate to avoid interference with bacteriological sampling. The enamel was removed with sterile high-speed fissure bur and the operation area was disinfected again. The initial entry to pulp space was made by sterile burs under manual irrigation with sterile 0.9% saline solution. The patency of the root canal was established by a sterile 15 K-type file without any usage of chemical solvents. Consecutively autoclave-sterilized dry paper points were inserted in the full length of the canal using the preoperative radiograph so that the fluid inside the root canal was absorbed. If the canal was dry, a little sterile 0.9% saline would be syringed into it first. The paper points per root canal were pooled into a 1.5 ml centrifuge tube containing 1 ml sterile pre-reduced PBS (Phosphate Buffered Saline) and transported to Shanghai Key Laboratory of Stomatology, China for culture within 20 min.

### Culture isolation

The clinical transport fluid mentioned above was diluted 100X and 50 μl dilution was inoculated into anaerobic blood agar CDC-based selective medium for black pigmented bacteria [72-74]. The content of the medium was 48 g CDC powder (Hangzhou Tianhe Microorganism Reagent Co., Ltd), 50 ml sheep blood, 1 ml Vitamin K1, 0.1 g kanamycin (Sangon Biotech (Shanghai) Co., Ltd) and 7.5 mg vancomycin (Japan, Eli Lily Japan KK) dissolved in 1 L distilled water. The plates were cultured in an anaerobic incubator (UK, Ruskinn) in an atmosphere of 85% N_2_, 10% CO_2_, 5% H_2_ at 37°C for 7 days. 95 black colonies appeared in 3 of the 13 samples and were all isolated for further purification by subculture. The pure colonies were picked into eppendorf tubes and stored at -20°C.

### PCR testing

Genome DNA of the 95 purified clones and standard strain *Prevotella intermedia* ATCC25611 supplied by Shanghai Key Laboratory of Stomatology, China were extracted with TIANamp Bacteria DNA Kit (Tiangen Biotech Co., LTD, Beijing, China) according to the manual. The DNA concentration of the samples is measured with spectrophotometer (Thermo Fisher Scientific Co., Ltd, Massachusetts, USA). The samples were diluted to 5 ng/μl and amplified with *Prevotella intermedia* specific PCR primers with the expected product length of 576 bp: the forward primer: 5′-TTTGTTGGGGAGTAAAGCGGG-3′; the reverse primer: 5′-TCAACATCTCTGTATCCTGCGT-3′ (Sangon Biotech Co., Ltd, Shanghai, China). 1 μl DNA template was added to the PCR mixture containing 12.5 μl Premix Ex Taq vention (Biotechnology Co., LTD, Dalian, China) and 0.5 μl of each primer and 10.5 μl sterilized ultrapure water. The PCR was carried out in a thermal cycle (Applied Biosystems, California, USA) under the program as follows: 94°C for 10 min, 35 cycles at 94°C for 1 min, 60°C for 30 s,72°C for 30 s, and a final 72°C for 7 min. PCR products were analyzed with 1% agarose gel electrophorisis. The agarose gel was stained by ethidium bromide, and the image of the gel was captured under UV light by CCD camera. The sample that yielded the predicted amplicon of 576 bp were predicted to belong to *Prevotella intermedia* species. They were selected and sent to Shanghai genomePilot Technology, Inc for whole-length 16 s rDNA sequencing. The DNA sample were applified with general 16 s DNA primer Forward 5′-AGAGTTTGATCCTGGCTCAG-3′, Reverse 5′-ACGGCTACCTTGTTACGACTT-3′. The Product is sequenced and compared against NCBI database with nBLAST tool. The result confirmed that the strains belonged to *Prevotella intermedia* species

### Genome sequencing, assembly and annotation

The genomic DNA was sequenced by Roche 454 GS FLX system according to the standard protocol provided by the manufacturer and using the default parameter. 309,368 reads totaling 123,745,995 nucleotides with an average length of 400 bp per read were generated, providing approximately 65X of genome coverage. The raw data was trimmed using SeqClean and Lucy (1.20p version) to eliminate joint sequences generated during library building and sequencing. 871 reads shorter than 50 bp were also left out. 167 contaminated reads from other species were detected by blasting against the NCBI NT Database and omitted from further analysis. 308,007 reads totaling 123,017,649 bp remained, 99.79% of which were assembled by Newbler 2.5.3 into 89 contigs amounting to 2,758,856 bp and 320 singletons amounting to 53,951 bp.

ORF prediction was performed using GLIMMER 3.0. The ORF sequences were *in silico* translated and search against Non-redundant protein sequences database with blastp with the threshold of e < 1e-3 and identity > 40%. Only the best hits were used for gene function prediction. tRNA, tmRNA, rRNA were predicted by tRNAscan-SE (http://lowelab.ucsc.edu/tRNAscan-SE/), ARAGORN and RNAmmer1.2 respectively.

Other genomes of the genus *Prevotella* were downloaded from NCBI ftp (ftp://ftp.ncbi.nih.gov/genomes/).

The predicted protein sequences were classified with on-line COG engine WebMGA [75], which aligned the query sequences to the COG database updated on Feb 2nd 2011 with rpsblast 2.2.15 [76].

The protein sequences were mapped to KEGG [59] pathway using the online server KAAS [77] (http://www.genome.jp/tools/kaas/).

### Genomic sequence comparison

The genome comparison was carried out using MUMmer 3.23. The analysis was performed according to the online manual (http://mummer.sourceforge.net/manual/) and the result was visualized by mapview.

Gene comparison was carried out using Blastn, with the threshold of e-value = 10^−10^, coverage = 80%, identity = 60%.

### Pan-genome analysis with PGAP

Pan-Genomes Analysis Pipeline (PGAP) is a perl-script pipeline that consists of orthologous gene clustering, pan-genome analysis, SNP in CDS calling, evolutionary analysis and COG distribution.

The orthologous gene clustering was performed with The GeneFamily Method and ran as the following: The genes were first aligned using BLASTP with the following threshold: e-value of 1e-10 and the score of 40. Only gene pairs of Bidirectional Best Hits results (the identity greater than 50 and coverage greater than 0.5 from both sides) were clustered together. The other analysis was performed using the default parameter provided by the pipeline.

### KEGG pathway analysis

The predicted ORFs were mapped to the KEGG pathway by KEGG Automatic Annotation Server (KAAS) [77]. The Genes data set was the default “for prokaryotes” and the assignment method was bi-directional best hit.

### Prediction of protease

We predicted genes for proteases by two methods: we 1) searched among the annotation of ORFs and 2) searched all the predicted ORFs against the MEROPS release 8.9 Protease Database. The 2 sets of results were then merged. The genes predicted to be protease by MEROPS but annotated to be enzymes other than protease with a high score and identity by BLAST were manually removed.

We further aligned all the predicted protease genes against the PSORTb [43] for the cellular location.

### Phylogenetics of the genus *Prevotella*

In order to infer the phylogenetic relationships of the *Prevotella intermedia* ZT strain and the other 35 *Prevotella* taxa (Additional file 3), we first downloaded the genome data from NCBI ftp (ftp://ftp.ncbi.nih.gov/). Then the putative orthologous gene sets were generated through the following process: we first compared the all the 2067 predicted translated ORF sequences of *Prevotella intermedia* ZT strain against other taxa of the genus *Prevotella* by BLASTP method with the cutoff e-value of 0.1. 717 genes queries that have at least one hit in every species were selected for further analysis. Every query and its best representations of each species were viewed as a set of putative homologous genes. From these gene sets we manually screened for those that were 1) annotated to have the same biological function and 2) the housekeeping genes which are not likely to be influenced by horizontal gene transfer. 80 gene sets were picked and aligned and by ClustalW2.1 [78] with default parameter. The alignment files were clipped and concatenated into a set of 34581-nt multi-gene sequence with the help of BioEdit7.0.

The phylogenetic trees were built with Maximum Likelihood and Neighbor joining method in the Molecular Evolutionary Genetics Analysis (MEGA5.10) [79]. For the ML method, Jones-Taylor-Thornton (JTT) model was used and the result was tested by bootstrap method with the bootstrap replications of 1,000. The rates among sites were set as uniform and all the gap and missing data were deleted. The NJ tree was generated using Poisson method and also tested by 1000-replication bootstrap.

### Availability of supporting data

The genome sequence data of *Prevotella intermedia* ZT (BioProject ID: PRJNA208776; accession: ATMK00000000; BioSample SAMN02212661) is available at NCBI http://www.ncbi.nlm.nih.gov/bioproject/208776.

## Additional files

Additional file 1:
**The clustered strain specific genes in** ***Prevotella intermedia*** **.**
Additional file 2:
**The visualized genomic alignments of** ***Prevotella*** **taxa.**
Additional file 3:
**The** ***Prevotella*** **taxa used in genome comparison and phylogenetics analysis.**
Additional file 4:
**The housekeeping genes used in phylogenetic tree building.**

## Abbreviations

CDS: Coding DNA Sequence
ORF: Open reading frame
LPS: Lipopolysaccharide
Pi: Prevotella intermedia
