# Supplementary figures and images for: Comparative genome analysis of Prevotella intermedia strain isolated from infected root canal reveals features related to pathogenicity and adaptation
Source: BMC Genomics. 2015 Feb 25;16(1):122. doi: 10.1186/s12864-015-1272-3 (PMC4349605; doi:10.1186/s12864-015-1272-3)

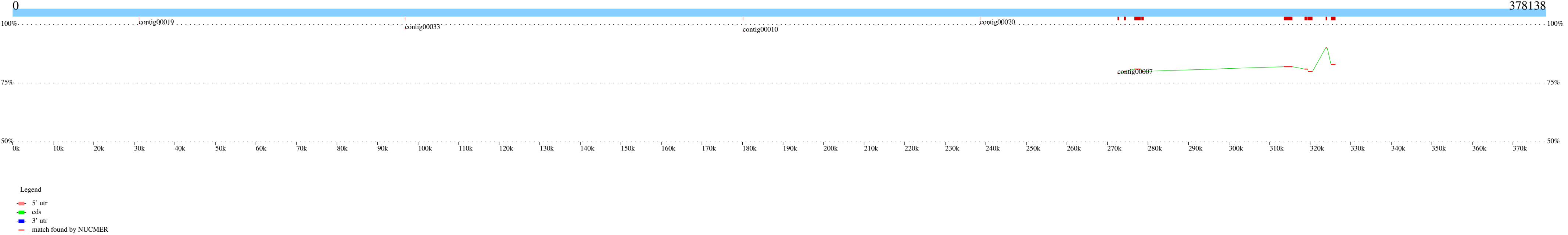

Supplement: Additional file 2: — The visualized genomic alignments of Prevotella taxa. [file 12864_2015_1272_MOESM2_ESM.zip › Prevotella_dentalis_DSM_3688_Contigs_0.pdf]

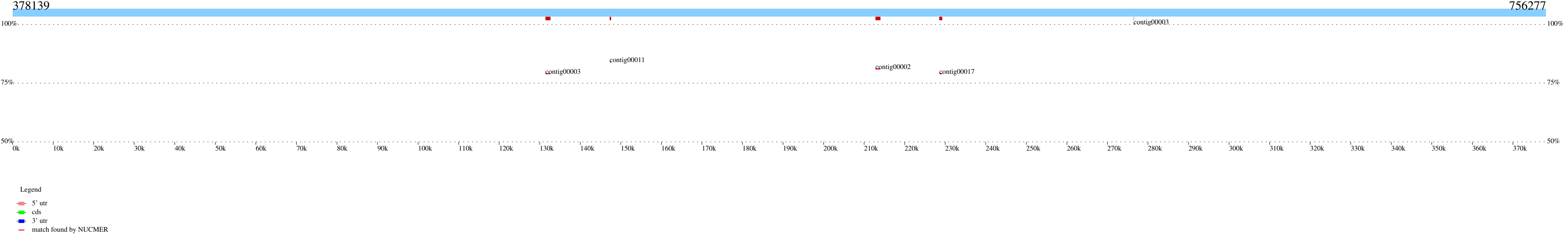

Supplement: Additional file 2: — The visualized genomic alignments of Prevotella taxa. [file 12864_2015_1272_MOESM2_ESM.zip › Prevotella_dentalis_DSM_3688_Contigs_1.pdf]

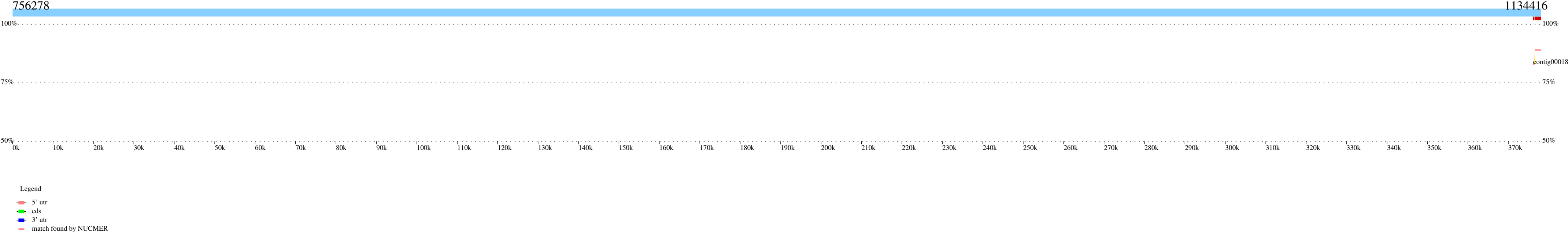

Supplement: Additional file 2: — The visualized genomic alignments of Prevotella taxa. [file 12864_2015_1272_MOESM2_ESM.zip › Prevotella_dentalis_DSM_3688_Contigs_2.pdf]

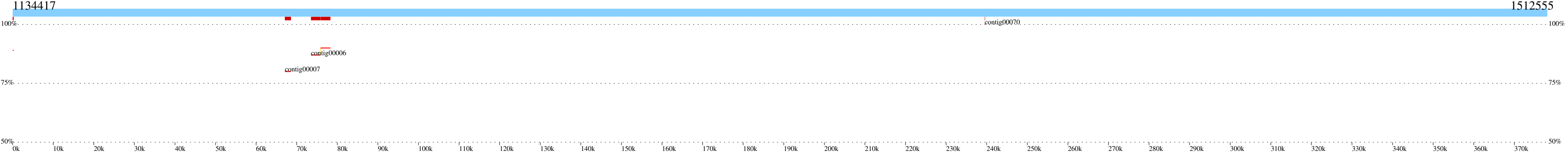

Legend

- 5' utr
- cds
- 3' utr
- match found by NUCMER

Supplement: Additional file 2: — The visualized genomic alignments of Prevotella taxa. [file 12864_2015_1272_MOESM2_ESM.zip › Prevotella_dentalis_DSM_3688_Contigs_3.pdf]

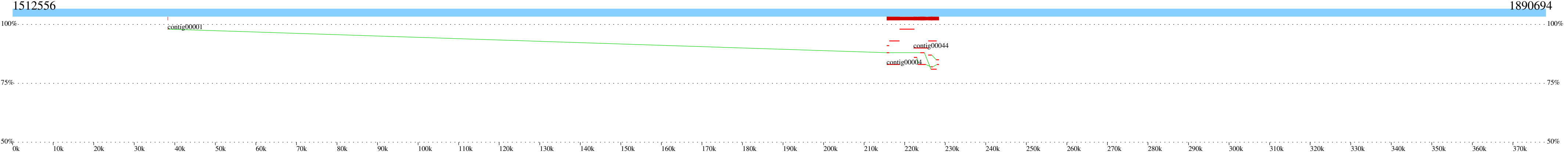

Supplement: Additional file 2: — The visualized genomic alignments of Prevotella taxa. [file 12864_2015_1272_MOESM2_ESM.zip › Prevotella_dentalis_DSM_3688_Contigs_4.pdf]

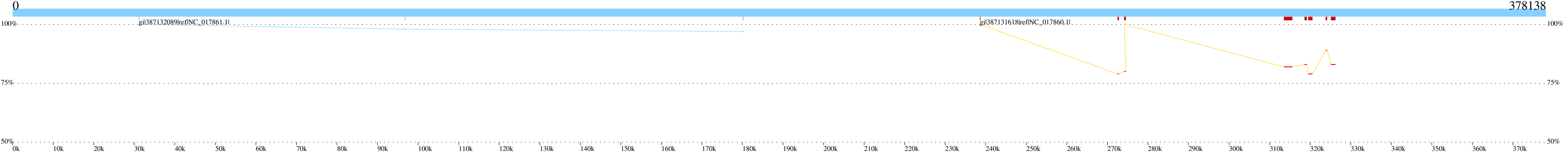

Supplement: Additional file 2: — The visualized genomic alignments of Prevotella taxa. [file 12864_2015_1272_MOESM2_ESM.zip › Prevotella_dentalis_DSM_3688_Prevotella_intermedia_17_0.pdf]

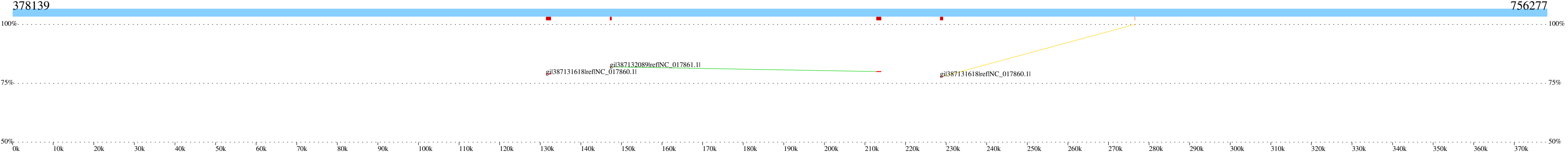

Supplement: Additional file 2: — The visualized genomic alignments of Prevotella taxa. [file 12864_2015_1272_MOESM2_ESM.zip › Prevotella_dentalis_DSM_3688_Prevotella_intermedia_17_1.pdf]

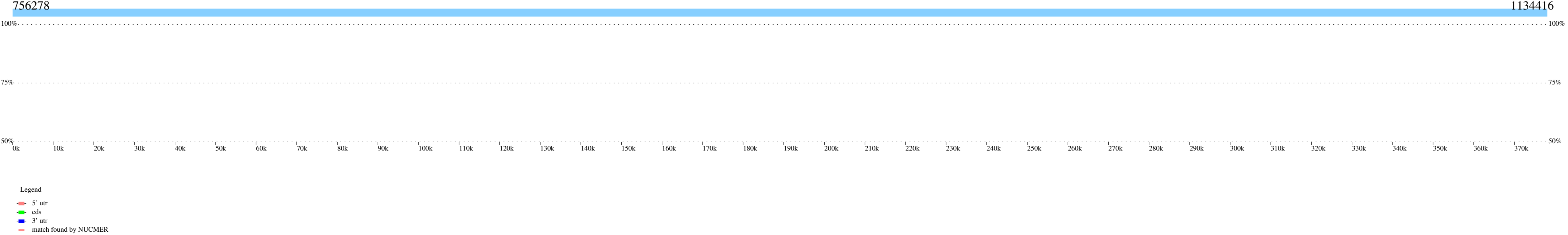

Supplement: Additional file 2: — The visualized genomic alignments of Prevotella taxa. [file 12864_2015_1272_MOESM2_ESM.zip › Prevotella_dentalis_DSM_3688_Prevotella_intermedia_17_2.pdf]

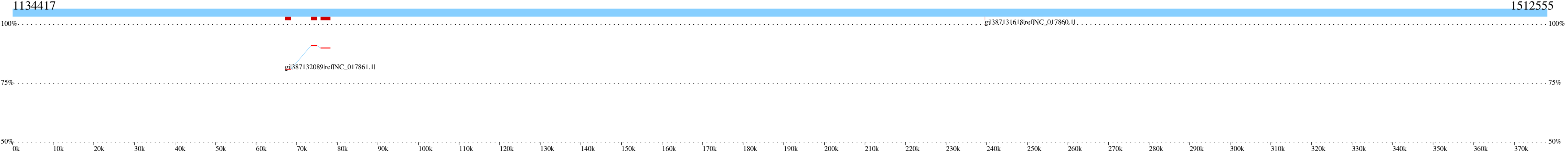

Supplement: Additional file 2: — The visualized genomic alignments of Prevotella taxa. [file 12864_2015_1272_MOESM2_ESM.zip › Prevotella_dentalis_DSM_3688_Prevotella_intermedia_17_3.pdf]

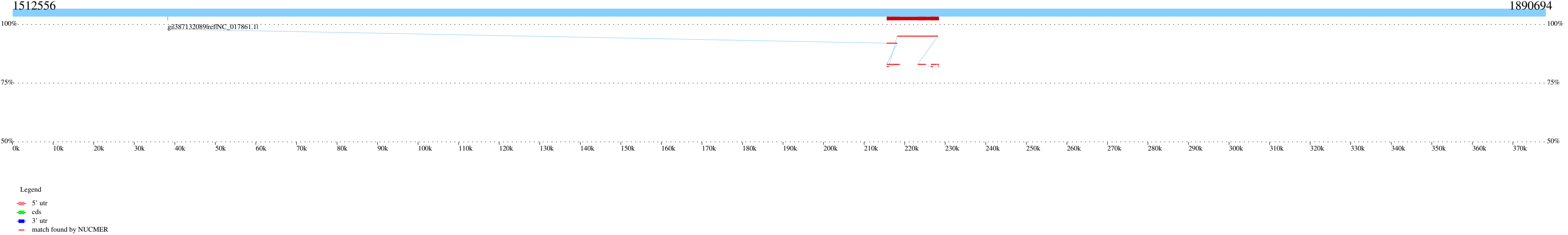

Supplement: Additional file 2: — The visualized genomic alignments of Prevotella taxa. [file 12864_2015_1272_MOESM2_ESM.zip › Prevotella_dentalis_DSM_3688_Prevotella_intermedia_17_4.pdf]

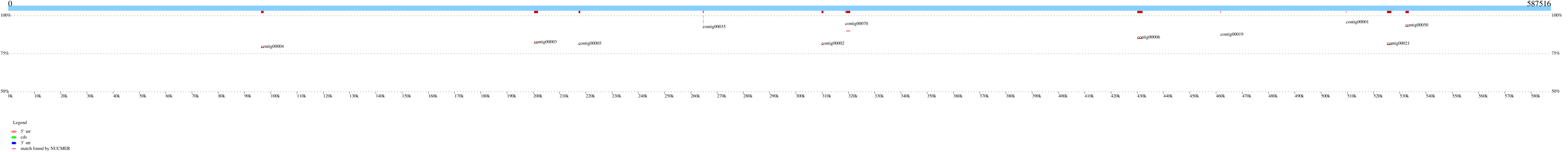

Supplement: Additional file 2: — The visualized genomic alignments of Prevotella taxa. [file 12864_2015_1272_MOESM2_ESM.zip › Prevotella_denticola_F0289_Contigs_0.pdf]

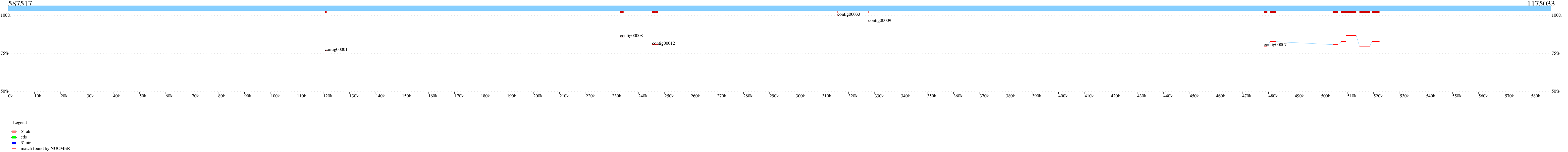

Supplement: Additional file 2: — The visualized genomic alignments of Prevotella taxa. [file 12864_2015_1272_MOESM2_ESM.zip › Prevotella_denticola_F0289_Contigs_1.pdf]

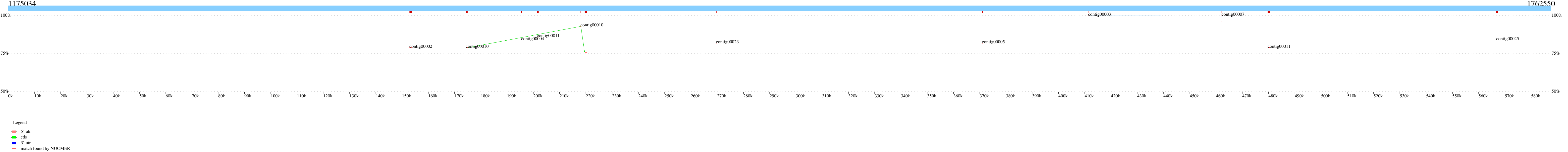

Supplement: Additional file 2: — The visualized genomic alignments of Prevotella taxa. [file 12864_2015_1272_MOESM2_ESM.zip › Prevotella_denticola_F0289_Contigs_2.pdf]

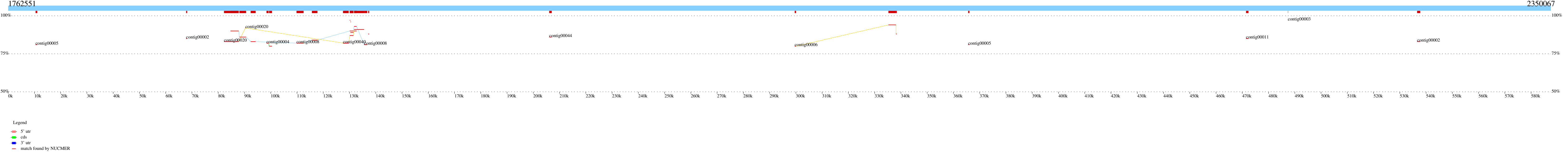

Supplement: Additional file 2: — The visualized genomic alignments of Prevotella taxa. [file 12864_2015_1272_MOESM2_ESM.zip › Prevotella_denticola_F0289_Contigs_3.pdf]

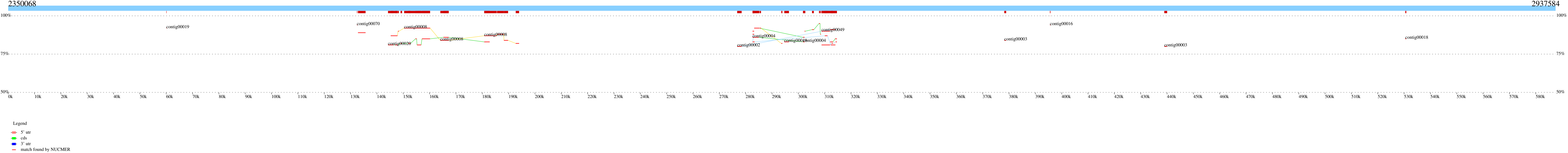

Supplement: Additional file 2: — The visualized genomic alignments of Prevotella taxa. [file 12864_2015_1272_MOESM2_ESM.zip › Prevotella_denticola_F0289_Contigs_4.pdf]

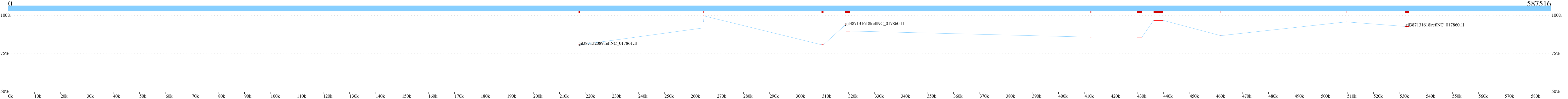

Legend

- 5' utr
- cds
- 3' utr
- match found by NUCMER

Supplement: Additional file 2: — The visualized genomic alignments of Prevotella taxa. [file 12864_2015_1272_MOESM2_ESM.zip › Prevotella_denticola_F0289_Prevotella_intermedia_17_0.pdf]

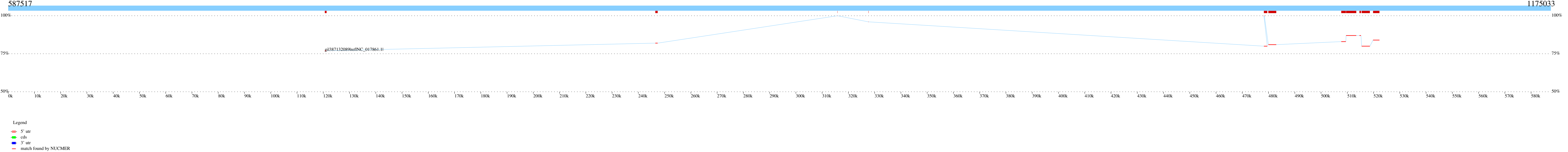

Supplement: Additional file 2: — The visualized genomic alignments of Prevotella taxa. [file 12864_2015_1272_MOESM2_ESM.zip › Prevotella_denticola_F0289_Prevotella_intermedia_17_1.pdf]

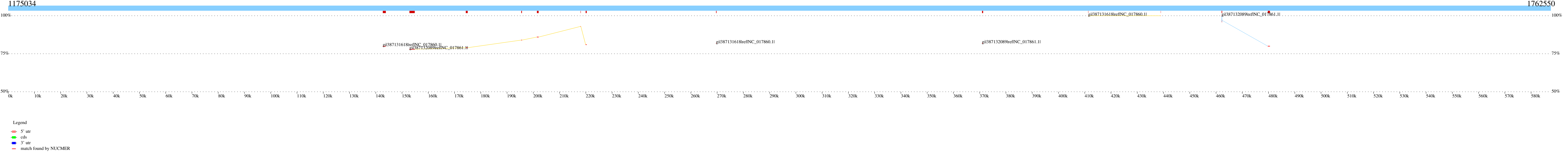

Supplement: Additional file 2: — The visualized genomic alignments of Prevotella taxa. [file 12864_2015_1272_MOESM2_ESM.zip › Prevotella_denticola_F0289_Prevotella_intermedia_17_2.pdf]

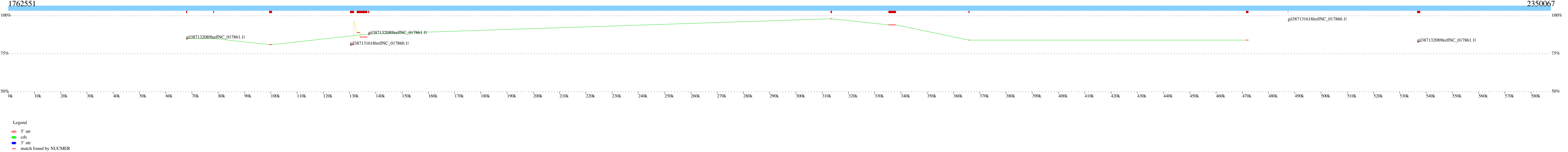

Supplement: Additional file 2: — The visualized genomic alignments of Prevotella taxa. [file 12864_2015_1272_MOESM2_ESM.zip › Prevotella_denticola_F0289_Prevotella_intermedia_17_3.pdf]

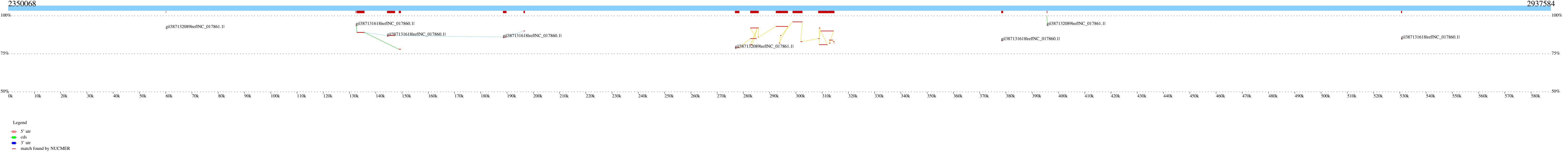

Supplement: Additional file 2: — The visualized genomic alignments of Prevotella taxa. [file 12864_2015_1272_MOESM2_ESM.zip › Prevotella_denticola_F0289_Prevotella_intermedia_17_4.pdf]

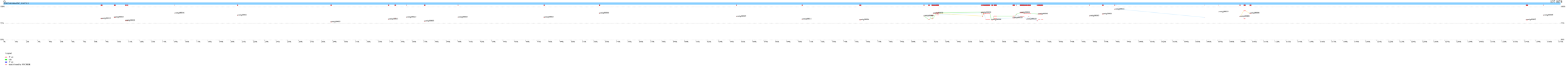

Supplement: Additional file 2: — The visualized genomic alignments of Prevotella taxa. [file 12864_2015_1272_MOESM2_ESM.zip › Prevotella_melaninogenica_ATCC_25845_Contigs_2.pdf]

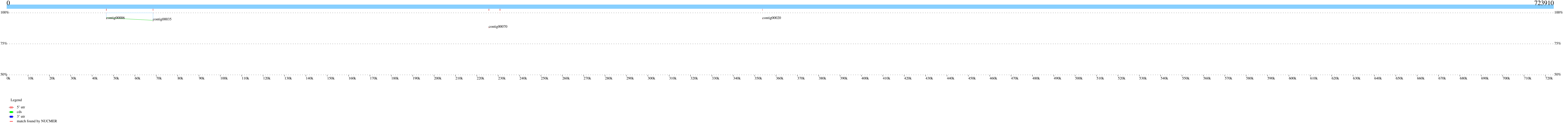

Supplement: Additional file 2: — The visualized genomic alignments of Prevotella taxa. [file 12864_2015_1272_MOESM2_ESM.zip › Prevotella_ruminicola_23_Contigs_0.pdf]

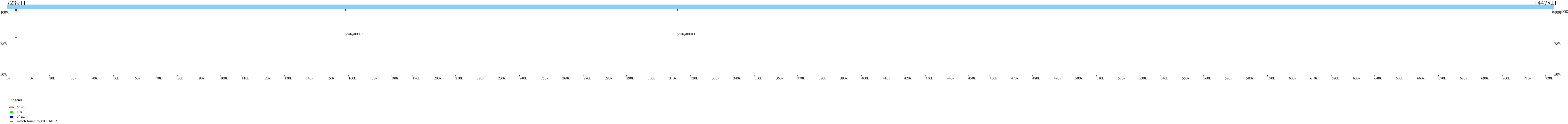

Supplement: Additional file 2: — The visualized genomic alignments of Prevotella taxa. [file 12864_2015_1272_MOESM2_ESM.zip › Prevotella_ruminicola_23_Contigs_1.pdf]

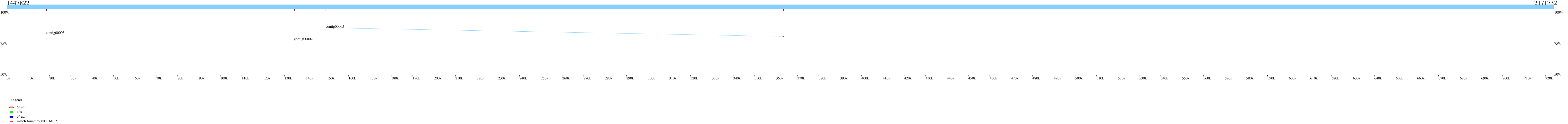

Supplement: Additional file 2: — The visualized genomic alignments of Prevotella taxa. [file 12864_2015_1272_MOESM2_ESM.zip › Prevotella_ruminicola_23_Contigs_2.pdf]

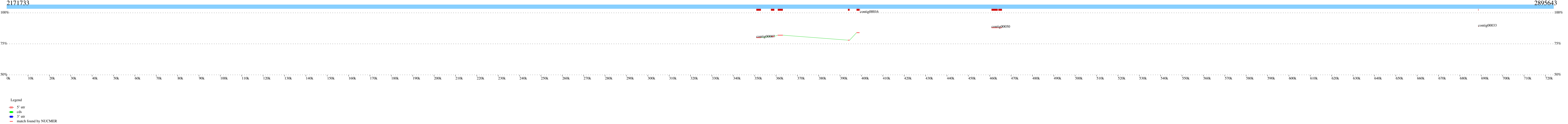

Supplement: Additional file 2: — The visualized genomic alignments of Prevotella taxa. [file 12864_2015_1272_MOESM2_ESM.zip › Prevotella_ruminicola_23_Contigs_3.pdf]

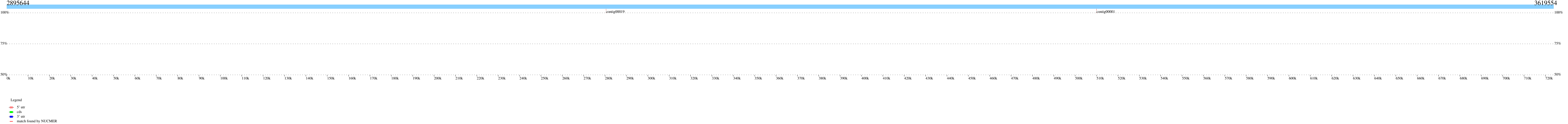

Supplement: Additional file 2: — The visualized genomic alignments of Prevotella taxa. [file 12864_2015_1272_MOESM2_ESM.zip › Prevotella_ruminicola_23_Contigs_4.pdf]

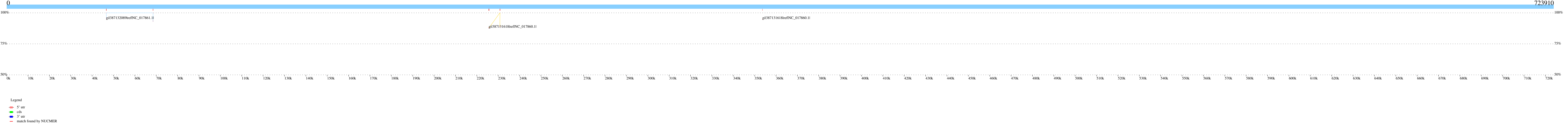

Supplement: Additional file 2: — The visualized genomic alignments of Prevotella taxa. [file 12864_2015_1272_MOESM2_ESM.zip › Prevotella_ruminicola_23_Prevotella_intermedia_17_0.pdf]

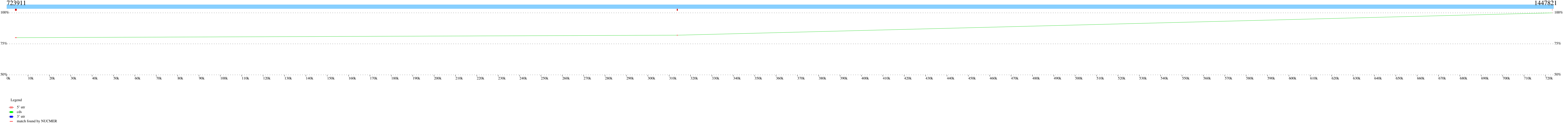

Supplement: Additional file 2: — The visualized genomic alignments of Prevotella taxa. [file 12864_2015_1272_MOESM2_ESM.zip › Prevotella_ruminicola_23_Prevotella_intermedia_17_1.pdf]

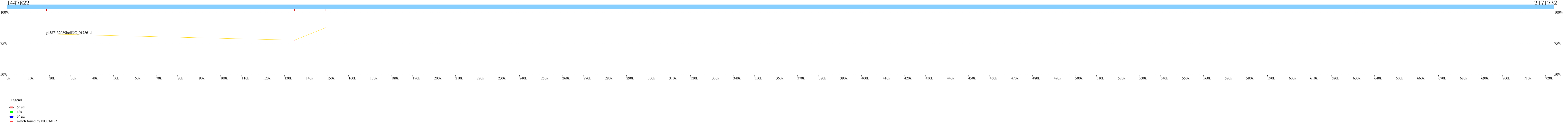

Supplement: Additional file 2: — The visualized genomic alignments of Prevotella taxa. [file 12864_2015_1272_MOESM2_ESM.zip › Prevotella_ruminicola_23_Prevotella_intermedia_17_2.pdf]

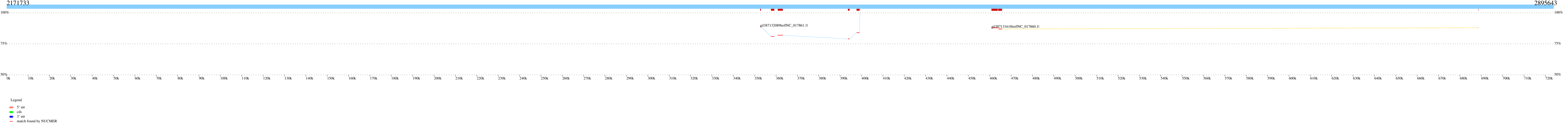

Supplement: Additional file 2: — The visualized genomic alignments of Prevotella taxa. [file 12864_2015_1272_MOESM2_ESM.zip › Prevotella_ruminicola_23_Prevotella_intermedia_17_3.pdf]

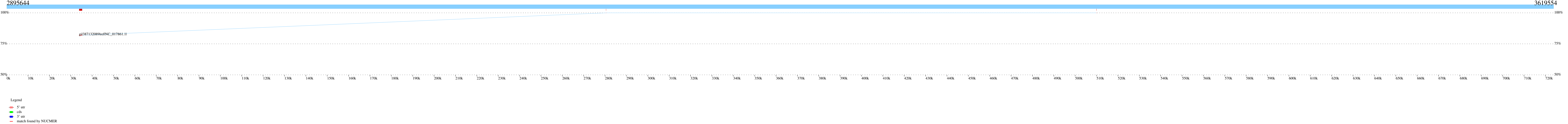

Supplement: Additional file 2: — The visualized genomic alignments of Prevotella taxa. [file 12864_2015_1272_MOESM2_ESM.zip › Prevotella_ruminicola_23_Prevotella_intermedia_17_4.pdf]
