# Supplementary material for: Comparative genome analysis of Prevotella intermedia strain isolated from infected root canal reveals features related to pathogenicity and adaptation
Source: BMC Genomics. 2015 Feb 25;16(1):122. doi: 10.1186/s12864-015-1272-3 (PMC4349605; doi:10.1186/s12864-015-1272-3)
Supplement: Additional file 2: — The visualized genomic alignments of Prevotella taxa. [file Prevotella_intermedia_17_Contigs_1.pdf › Prevotella_intermedia_17_Contigs_1.pdf]

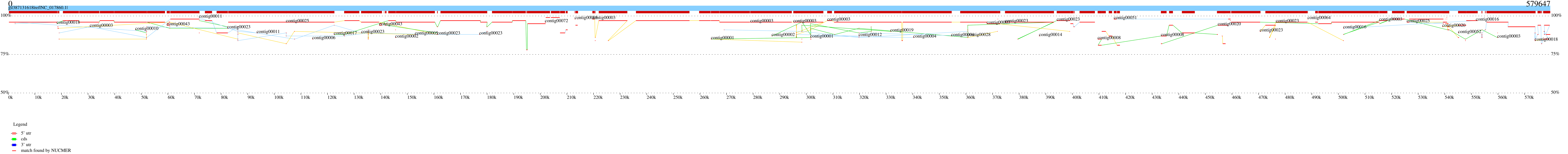
0
579647
gi|387131618|ref|NC_017860.1|
100%
75%
50%
0k
10k
20k
30k
40k
50k
60k
70k
80k
90k
100k
110k
120k
130k
140k
150k
160k
170k
180k
190k
200k
210k
220k
230k
240k
250k
260k
270k
280k
290k
300k
310k
320k
330k
340k
350k
360k
370k
380k
390k
400k
410k
420k
430k
440k
450k
460k
470k
480k
490k
500k
510k
520k
530k
540k
550k
560k
570k
Legend
5' utr
cds
3' utr
match found by NUCMER
