# Supplementary material for: Comparative genome analysis of Prevotella intermedia strain isolated from infected root canal reveals features related to pathogenicity and adaptation
Source: BMC Genomics. 2015 Feb 25;16(1):122. doi: 10.1186/s12864-015-1272-3 (PMC4349605; doi:10.1186/s12864-015-1272-3)
Supplement: Additional file 2: — The visualized genomic alignments of Prevotella taxa. [file Prevotella_melaninogenica_ATCC_25845_Contigs_1.pdf › Prevotella_melaninogenica_ATCC_25845_Contigs_1.pdf]

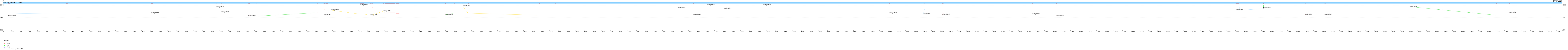
0
gi|302344773|ref|NC_014370.1|
100%
contig00001
contig00012
75%
50%
Legend
5’ utr
cds
3’ utr
match found by NUCMER
