# Supplementary material for: Comparative genome analysis of Prevotella intermedia strain isolated from infected root canal reveals features related to pathogenicity and adaptation
Source: BMC Genomics. 2015 Feb 25;16(1):122. doi: 10.1186/s12864-015-1272-3 (PMC4349605; doi:10.1186/s12864-015-1272-3)
Supplement: Additional file 2: — The visualized genomic alignments of Prevotella taxa. [file Prevotella_melaninogenica_ATCC_25845_Prevotella_intermedia_17_1.pdf › Prevotella_melaninogenica_ATCC_25845_Prevotella_intermedia_17_1.pdf]

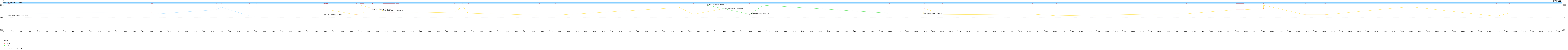

0
gi|302344773|ref|NC_014370.1|
1796408
100%
75%
50%
gi|387132089|ref|NC_017861.1|
0k
10k
20k
30k
40k
50k
60k
70k
80k
90k
100k
110k
120k
130k
140k
150k
160k
170k
Legend
5' utr
cds
3' utr
match found by NUCMER
