# Supplementary material for: Comparative genome analysis of Prevotella intermedia strain isolated from infected root canal reveals features related to pathogenicity and adaptation
Source: BMC Genomics. 2015 Feb 25;16(1):122. doi: 10.1186/s12864-015-1272-3 (PMC4349605; doi:10.1186/s12864-015-1272-3)
Supplement: Additional file 2: — The visualized genomic alignments of Prevotella taxa. [file Prevotella_melaninogenica_ATCC_25845_Prevotella_intermedia_17_2.pdf › Prevotella_melaninogenica_ATCC_25845_Prevotella_intermedia_17_2.pdf]

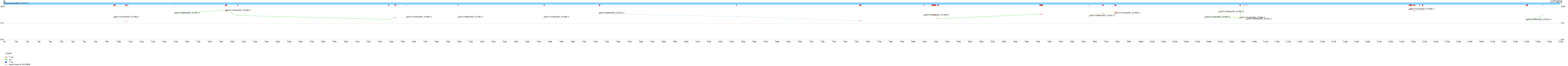
0
1371874
gi|302346166|ref|NC_014371.1|
100%
75%
50%
gi|387131618|ref|NC_017860.1|
gi|387132089|ref|NC_017861.1|
Legend
5' utr
cds
3' utr
match found by NUCMER
